# Publisher Correction: The complexity of NISQ

**DOI:** 10.1038/s41467-024-45799-7

**Published:** 2024-02-12

**Authors:** Sitan Chen, Jordan Cotler, Hsin-Yuan Huang, Jerry Li

**Affiliations:** 1 Department of Electrical Engineering and Computer Science, University of California, Berkeley, Berkeley, CA USA; 2 Society of Fellows, Harvard University, Cambridge, MA USA; 3 Institute for Quantum Information and Matter, CAltech, Pasadena, CA USA; 4 Department of Computing and Mathematical Sciences, CAltech, Pasadena, CA USA; 5 Microsoft Research AI, Redmond, WA USA; 6 Present Address: John A. Paulson School of Engineering and Applied Sciences, Harvard University, Cambridge, USA

**Keywords:** Quantum information, Computer science, Information theory and computation

Correction to: *Nature Communications* 10.1038/s41467-023-41217-6, published online 26 September 2023

In the original pdf version of this Article, the text of Theorems 2.2 and 2.3 in the Results section was missing the ⊊ symbol. The html version displayed the theorems in the correct form.

This has now been corrected.

