# Supplementary material for: The complexity of NISQ
Source: Nat Commun. 2023 Sep 26;14:6001. doi: 10.1038/s41467-023-41217-6 (PMC10522708; doi:10.1038/s41467-023-41217-6)
Supplement: Supplementary file 1 — Supplementary Information [file 41467_2023_41217_MOESM1_ESM.pdf]

Supplementary Information
The Complexity of NISQ

Sitan Chen, Jordan Cotler, Hsin-Yuan Huang, Jerry Li

Supplementary Note 1 – Related Work

In this section, we review several related topics in the literature.

Faulty oracle models. A number of works have studied the effect of *imperfect oracles* on quantum speedups. For instance, [1] studied whether the exponential speedup achieved by the quantum annealing algorithm for the welded tree problem persists when the oracle is subjected to various kinds of noise. Relevant to our Theorem 2.4, [2, 3] considered the performance of Grover's algorithm when the phase oracle is subject to small phase fluctuations, and [4] showed that under this faulty oracle model speedups are not possible for any quantum algorithm; see also [5] for a different oracle noise model. Relevant to our Theorem 2.5, [6, 7, 8] showed that noise in an oracle for subset parity does not affect the computational complexity of quantum learning algorithms in the same way conjectured for classical learning algorithms.

These results assume that noise occurs inside the oracle, but that the quantum computation leveraging the faulty oracle is noiseless. Moreover, the lower bounds in [1, 4, 5] assume a global noise on the oracle as opposed to local qubit-wise noise considered in this work. In contrast, we study the effects of imperfections in quantum computation due to local noise, as well as noisy oracles with noise appearing before and after the oracle.

Noiseless hybrid quantum-classical models. A number of works have studied the power and limitations of hybrid quantum-classical models when the quantum computation is assumed to be noiseless. For example, the recent work [9] studies unstructured search in a noiseless hybrid setting where the algorithm can make queries to both classical and quantum versions of the search oracle. They show that any algorithm with constant success probability must make either $\Omega(\sqrt{N})$ queries to the quantum oracle or $\Omega(N)$ queries to the classical oracle. Earlier works [10, 11] showed that relative to various oracles, namely the recursive Simon's and welded tree problem, BQP is strictly more powerful than classical computation, which is assisted by a noiseless bounded-depth quantum device. The oracle we use in Theorem 2.3 is essentially a simplified version of the recursive Simon's problem, and in Supplementary Note 8, we show how recursive Simon's itself can be used to simultaneously separate NISQ and the complexity classes considered in [10, 11] from BQP. In [12], the authors used a very different proof technique to establish a statement that is equivalent to our Theorem 5.B.1, which is the main component for establishing Theorem 2.4 on the lack of Grover-like quadratic speedup in NISQ on unstructured search. [12] uses an approach based on potential function, while we use a technique for proving lower bounds for learning quantum states and processes with unentangled measurements. Moreover, the connection between lower bounds for bounded-depth quantum computation and lower bounds for NISQ is new to our work.

Noise resilience of specific algorithms. A number of works have studied how existing algorithms perform under various forms of noise in their implementation. For unstructured search, [13, 14] studied whether Grover's algorithm is robust to various deviations like noisy Hadamard

gates and Gaussian noise between iterations, while [15] demonstrated that recursive amplitude amplification is robust to noisy reflection operators. [16, 17, 18, 19, 20], among others, studied the resilience of specific quantum optimization algorithms like QAOA and VQE under models similar to our definition of λ-noisy circuits. [20] found that in various regimes, these algorithms suffer significant slowdown when implemented on noisy quantum devices due to the flattening of the cost landscape. [21] shows that estimating the output probability of random quantum circuits to exponentially small additive error remains #P-hard even under the presence of small noise. Furthermore, [16] showed that the presence of noise can make these quantum algorithms easy to simulate on classical computers. In contrast, in the present work, we study the capabilities and limitations for NISQ without necessarily focusing on any particular algorithm.

Complexity of noisy quantum circuits. To our knowledge, the only other paper to study the relation of noisy quantum circuits and existing complexity classes is that of [22], which considered a generalization of our notion of λ-noisy circuits in which a random fraction of qubits at every layer are adversarially corrupted. Notably, they showed that polynomial-size noisy quantum circuits are no stronger than the complexity class QNC^1 of logarithmic-depth noiseless circuits, whereas quasipolynomial-size noisy quantum circuits can compute any function in QNC^1. Recall that in the present work, rather than study λ-noisy circuits in isolation, we consider the power of classical computation augmented by such circuits.

Supplementary Note 2 – The NISQ Complexity Class

In this section, we formally define the complexity class NISQ. Then we recall the notion of classical oracles in classical (BPP) and quantum computation (BQP) and generalize this to NISQ.

2.A Definition of the complexity class

We begin by recalling the single-qubit depolarizing channel D_λ.

Definition 2.A.1 (Single-qubit depolarizing channel). *Given $\lambda \in [0, 1]$. We define the* single-qubit depolarizing channel *to be $D_\lambda[\rho] \triangleq (1-\lambda)\rho + \lambda(I/2)$, where ρ is a single-qubit density matrix.*

Definition 2.A.2 (Depth-1 unitary). *Given $n > 0$. An n-qubit unitary U is a* depth-1 unitary *if U can be written as a tensor product of two-qubit unitaries.*

We consider noisy quantum circuits with noise level λ to be defined as follows.

Definition 2.A.3 (Output of a noisy quantum circuit). *Let $\lambda \in [0, 1]$ and $n \in \mathbb{N}$. Given $T \in \mathbb{N}$ and a sequence of T depth-1 unitaries $U_1, \ldots, U_T$, the output of the corresponding* λ-noisy depth-T quantum circuit *is a random n-bit string $s \in \{0, 1\}^n$ sampled from the distribution*

$$p(s) = \langle s| D_\lambda^{\otimes n}\left[U_T \ldots D_\lambda^{\otimes n}\left[U_2 D_\lambda^{\otimes n}\left[U_1 D_\lambda^{\otimes n}[|0^n\rangle\langle 0^n|]U_1^\dagger\right]U_2^\dagger\right] \ldots U_T^\dagger\right] |s\rangle\ , \tag{1}$$

where every quantum operation is followed by a layer of single-qubit depolarizing channel. When $\lambda = 0$, we say that this circuit is noiseless.

Remark 2.A.1. *We work with the single-qubit depolarizing channel as it is the most standard model for local noise. One could also consider stronger noise models, e.g. every qubit is randomly corrupted with probability λ by an adversary rather than randomly decohered. Tautologically, the lower bounds we prove in this work will translate to such stronger models. We also prove our upper bounds, namely Theorem 2.2 and 2.5, under this stronger model (see Remarks 4.A.1 and 6.B.1).*

Definition 2.A.4 (Noisy quantum circuit oracle)**.** *We define* NQC_λ *to be an oracle that takes in an* $n \in \mathbb{N}$ *and a sequence of depth-*1 *n-qubit unitaries* $\{U_k\}_{k=1,\ldots,T}$ *for any* $T \in \mathbb{N}$ *and outputs a random n-bit string s according to Eq.* (1)*.*

We define the time to query NQC_λ *with* T *depth-*1 *n-qubit unitaries to be* $\Theta(nT)$*, which is linear in the time to write down the input to the query.*

We now define NISQ algorithms, which are classical algorithms with access to the noisy quantum circuit oracle. This provides a formal definition for hybrid noisy quantum-classical computation.

Definition 2.A.5 (NISQ algorithm)**.** *A* NISQ_λ algorithm with access to λ-noisy quantum circuits *is defined as a probabilistic Turing machine* M *that can query* NQC_λ *to obtain an output bitstring s for any number of times, and is denoted as* $A_\lambda \triangleq M^{\mathrm{NQC}_\lambda}$*. The runtime of* A_λ *is given by the classical runtime of* M *plus the sum of the times to query* NQC_λ*.*

The NISQ complexity class for decision problems is defined as follows. Observe that the following recovers the definition for BPP when M^{NQC_λ} in the definition of A_λ above is replaced by M.

Definition 2.A.6 (NISQ complexity)**.** *A language* $L \subseteq \{0,1\}^*$ *is in* NISQ *if there exists a* NISQ_λ *algorithm* A_λ *for some constant* $\lambda > 0$ *that decides* L *in polynomial time, that is, such that*

- *for all* $x \in \{0,1\}^*$, A_λ *produces an output in time* $\mathrm{poly}(|x|)$*, where* $|x|$ *is the length of* x*;*
- *for all* $x \in L$, A_λ *outputs* 1 *with probability at least* 2/3*;*
- *for all* $x \notin L$, A_λ *outputs* 0 *with probability at least* 2/3*.*

Remark 2.A.2. *The noise parameter* λ *in our definition of* NISQ *is taken to be an absolute constant. One can also consider variants in which* λ *depends on the input length, or equivalently on the system size of the noisy quantum circuits. Note that if* λ *is a sufficiently quickly decaying function in these parameters, then the resulting complexity class will be equivalent to* BQP*. For instance, if* $\lambda \ll 1/N$ *where* N *is an upper bound on the width times depth of any noisy quantum circuit call, then with high probability, no noise gets applied over the course of the quantum computation. It is an interesting direction to explore how the complexity landscape mapped out in this paper changes in the intermediate regime where* λ *is only mildly decaying in the input length.*

2.B Algorithms with oracle access

In this work we study the complexity of learning a classical oracle or testing a property thereof. For instance, the unstructured search problem considers learning a classical oracle that highlights an element among N elements. We recall the following definition of a classical oracle O, as well as definitions of classical/quantum algorithms with access to the classical oracle O.

Definition 2.B.1 (Classical oracle O)**.** *A* classical oracle O *is a function from* $\{0,1\}^n$ *to* $\{0,1\}^m$ *for some* $n, m \in \mathbb{N}$*. The* $(n+m)$*-qubit unitary* U_O *corresponding to the classical oracle* O *is given by* $U_O |x\rangle |y\rangle = |x\rangle |y \oplus O(x)\rangle$ *for all* $x \in \{0,1\}^n, y \in \{0,1\}^m$*.*

Definition 2.B.2 (Classical algorithm with access to O)**.** *A* classical algorithm M^O with access to O *is a probabilistic Turing machine* M *that can query* O *by choosing an n-bit input* x *and obtaining the m-bit output* $O(x)$*.*

Definition 2.B.3 (Quantum algorithm with access to O)**.** *A* quantum algorithm Q^O with access to O *is a uniform family of quantum circuits* $\{U_n\}_n$*, where* U_n *is an* n'*-qubit quantum circuit given by*

$$U_n \triangleq V_{n,k}(U_O \otimes I) \cdots (U_O \otimes I) V_{n,2} (U_O \otimes I) V_{n,1} \, ,$$

for some integer $k \in \mathbb{N}$ *and* n'*-qubit unitaries* $V_{n,1}, \ldots, V_{n,k}$ *given as the product of many depth-*1 *unitaries. Here,* I *denotes the identity matrix over* $n' - n$ *qubits.*

We now present the definition of NISQ algorithms with access to the classical oracle O, which requires first defining noisy quantum circuit oracles with access to O.

Definition 2.B.4 (Noisy quantum circuit oracle with access to O)**.** *We define* NQC_λ^O *to be an oracle that takes in an integer* n' *and a sequence of* n'*-qubit unitaries* $\{U_k\}_{k=1,\ldots,T}$ *for any* $T \in \mathbb{N}$*, where* U_k *can either be a depth-*1 *unitary or* $U_O \otimes I$*, to a random* n*-bit string* s *sampled according to the distribution*

$$p(s) = \langle s| \, \mathcal{D}_\lambda^{\otimes n'} \left[U_T \ldots \mathcal{D}_\lambda^{\otimes n'} \left[U_2 \mathcal{D}_\lambda^{\otimes n'} \left[U_1 \mathcal{D}_\lambda^{\otimes n'} [|0^{n'}\rangle\langle 0^{n'}|] U_1^\dagger \right] U_2^\dagger \right] \ldots U_T^\dagger \right] |s\rangle \, .$$

Definition 2.B.5 (NISQ algorithm with access to O)**.** *Let* $\lambda \in [0,1]$*. A* NISQ_λ *algorithm* $A_\lambda^O = (M^{\mathrm{NQC}_\lambda})^O$ *with access to* O *is a probabilistic Turing machine* M *that has the ability to classically query* O *by choosing the* n*-bit input* x *to obtain the* m*-bit output* $O(x)$*, as well as the ability to query* NQC_λ^O *by choosing* n' *and* $\{U_k\}_{k=1,\ldots,T}$ *to obtain a random* n'*-bit string* s*. The runtime of* A_λ^O *is given by the sum of the classical runtime of* M*, the number of classical queries to* O*, and the sum of the times to query* NQC_λ^O*.*

With this definition in hand, we can extend the usual notions of relativized complexity to NISQ:

Definition 2.B.6 (Relativized NISQ)**.** *Given a sequence of oracles* $O : \{0,1\}^n \to \{0,1\}^{m(n)}$ *parametrized by* $n \in \mathbb{N}$*, a language* $L \subseteq \{0,1\}^*$ *is in* NISQ^O *if there exists a constant* $\lambda > 0$ *and a* NISQ_λ *algorithm* A_λ^O *with access to* O *that decides* L *in polynomial time.*

2.C Algorithms of bounded depth

In parts of this work we leverage the well-known connection [22] between noisy quantum circuits and noiseless bounded-depth circuits. Here we briefly recall some standard notions regarding the latter, presented in the language of Supplementary Note 2.A.

Definition 2.C.1 (Noiseless hybrid quantum-classical computation of bounded depth)**.** *A* noiseless depth-T algorithm *is a* NISQ_0 *algorithm* A *that only queries* NQC_0 *on sequences of depth-1* n*-qubit unitaries* $\{U_k\}_{k=1,\ldots,T'}$ *for* $1 \leq T' \leq T$*.*

Definition 2.C.2 ($\mathsf{BPP}^{\mathsf{QNC}}$)**.** *Let* $f : \mathbb{N} \to \mathbb{N}$ *be a nondecreasing function. A language* $L \subseteq \{0,1\}^*$ *is in* $\mathsf{BPP}^{\mathsf{QNC}[f(n)]}$ *if there is a noiseless depth-*$f(n)$ *algorithm* A *that decides* L *in polynomial time. When* $f(n) = O(\log^i(n))$*, we denote this class by* $\mathsf{BPP}^{\mathsf{QNC}^i}$*. We also define* $\mathsf{BPP}^{\mathsf{QNC}} \triangleq \cup_{i \geq 0} \mathsf{BPP}^{\mathsf{QNC}^i}$*.*

Note that $\mathsf{BPP}^{\mathsf{QNC}}$ is contained in the class BQP, as BQP can implement arbitrary *polynomial*-depth quantum computation.

We can also define noiseless depth-T algorithms with access to a classical oracle, as well as relativized versions of $\mathsf{BPP}^{\mathsf{QNC}^i}$ which we denote by $(\mathsf{BPP}^{\mathsf{QNC}^i})^O$, completely analogously to what is done in Section 2.B.

Supplementary Note 3 – Preliminaries

In this section, we summarize the basic mathematical techniques and ideas used in this work.

3.A Learning tree formalism and Le Cam's method

We begin by recalling the learning tree formalism of [23], adapted here to the setting of NISQ. This formalism will feature heavily in the proofs of our lower bounds against NISQ.

Definition 3.A.1 (Tree representation for NISQ algorithms)**.** *Given oracle $O : \{0,1\}^n \to \{0,1\}^m$, a NISQ_λ algorithm with access to O can be associated with a pair $(\mathcal{T}, \mathcal{A})$ as follows. The* learning tree *$\mathcal{T}$ is a rooted tree, where each node in the tree encodes the transcript of all classical query and noisy quantum circuit results the algorithm has seen so far. The tree satisfies the following properties:*

- *Each node u is associated with a value $p_O(u)$ corresponding to the probability that the transcript observed so far is given by the path from the root r to u. In this way, $\mathcal{T}$ naturally induces a distribution over its leaves. For the root r, $p_O(r) = 1$.*
- *At each non-leaf node u, we either classically query the oracle O at an input $x \in \{0,1\}^n$, or run a λ-noisy quantum circuit A with access to O.*
  - *(i) Classical query: u has a single child node v connected via an edge $(u, x, O(x))$, and we define*
  $$p_O(v) = p_O(u)\,.$$
  - *(ii) Noisy circuit query: The children v of u are indexed by the possible $s \in \{0,1\}^{n'}$ that could be obtained as a result. We refer to the edge between u and v as (u, A, s). We denote by $|\phi_O(A)\rangle$ the output state of the circuit so that the probability of traversing (u, A, s) from node u to child v is given by $|\langle s|\phi_O(A)\rangle|^2$. We define*
  $$p_O(v) = p_O(u) \cdot |\langle s|\phi_O(A)\rangle|^2\,.$$
- *If the total number of classical/quantum queries to O made along any root-to-leaf path is at most N, we say that the* query complexity *of the algorithm is at most N.*

$\mathcal{A}$ is any classical algorithm that takes as input a transcript corresponding to any leaf node ℓ and attempts to determine the underlying oracle or predict some property thereof.

The following lemma shows that slight perturbations to the distributions over children for each node do not change the overall distribution over leaves of $\mathcal{T}$ by too much.

Lemma 3.A.1. *Given learning tree $\mathcal{T}$ corresponding to a NISQ_λ algorithm with query complexity N, suppose $\mathcal{T}'$ is a learning tree obtained from $\mathcal{T}$ as follows. For every node u at which a noisy quantum circuit A is run, replace A by another circuit A' such that the new induced distribution over children of u is at most ε-far from the original distribution in total variation. Then the distributions over leaves of $\mathcal{T}$ and $\mathcal{T}'$ are at most εN-far in total variation.*

Proof. Consider the sequence of trees $\mathcal{T}^{(i)}$ where $\mathcal{T}^{(0)} = \mathcal{T}$ and $\mathcal{T}^{(i)}$ is given by taking all u in layer i of $\mathcal{T}^{(i-1)}$ that run some noisy quantum circuit A and replacing them with the corresponding circuit A' from $\mathcal{T}'$. By design, $\mathcal{T}^{(N)} = \mathcal{T}'$. Let $p^{(i)}$ denote the distribution over leaves of $\mathcal{T}^{(i)}$. It suffices to show that $d_{\mathrm{TV}}(p^{(i)}, p^{(i-1)}) \le \varepsilon$.

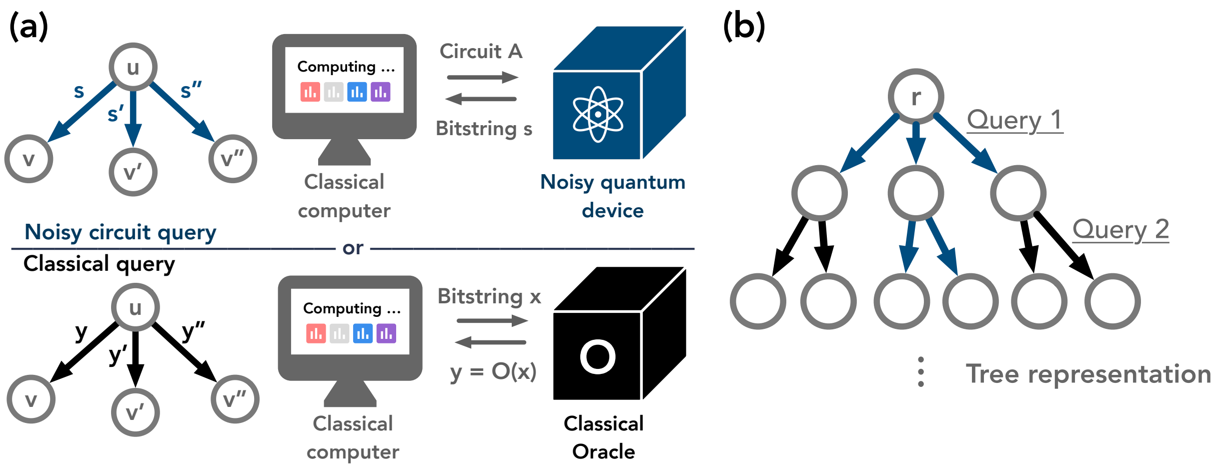

Supplementary Figure 1: Illustration of the tree representation for NISQ algorithms. (a) At every memory state u of the classical computer/algorithm, it could either make a noisy circuit query or a classical query. (b) The tree representation with a mix of noisy circuit queries and classical queries.

Note that $p^{(i-1)}$ specifies some mixture over distributions p_v, where p_v is the distribution over leaves conditioned on reaching node v in the i-th layer. In particular, in this mixture, v is sampled by sampling parent node u by running the NISQ algorithm corresponding to $\mathcal{T}'$ for $i-1$ steps and then running the corresponding quantum circuit A from $\mathcal{T}$. In contrast, $p^{(i)}$ is a mixture over the same distributions p_v, but v is sampled by running the NISQ algorithm corresponding to $\mathcal{T}'$ for i steps and then running the corresponding quantum circuit A' from $\mathcal{T}'$. These two distributions over v are at most ε-far in total variation, so the two mixture distributions are also at most ε-far in total variation as claimed. □

Our lower bounds will be based on Le Cam's method– see Section 4.3 of [23] for an overview in the context of the tree formalism of Definition 3.A.1. In every case we will reduce to some *distinguishing task* in which the algorithm must discern whether the oracle it has access to comes from one family of oracles or from another. For example, for unstructured search, the distinguishing task will be whether the oracle corresponds to some element in the search domain or whether the oracle is the identity channel.

More concretely, given two disjoint sets of oracles S_0, S_1, we will design distributions D_0, D_1 over S_0, S_1. Given any algorithm specified by some $(\mathcal{T}, \mathcal{A})$, we will upper bound the total variation distance between the following two distributions. We consider the mixture of distributions p_{O_i} over leaves of the learning tree when the underlying oracle O_i is sampled according to D_0 at the outset, as well as the mixture when the oracle is sampled according to D_1. The following lemma shows that upper bounding $d_{\mathrm{TV}}(\mathbb{E}_{i\sim D_0}[p_{O_i}], \mathbb{E}_{i\sim D_1}[p_{O_i}])$ suffices to show a query complexity lower bound for the distinguishing task:

Lemma 3.A.2 (Le Cam's two-point method, see e.g. Lemma 4.14 from [23]). *Let $\{O_i\}_{i\in S_0}$ and $\{O_i\}_{i\in S_1}$ be two disjoint sets of oracles. Given a tree $\mathcal{T}$ as in Definition 3.A.1 corresponding to a NISQ algorithm that makes N oracle queries, let p_i denote the induced distribution over leaves when the algorithm has access to O_i. If $d_{TV}(\mathbb{E}_{i\sim D_0}[p_0], \mathbb{E}_{i\sim D_1}[p_1]) < 1/3$, there is no algorithm $\mathcal{A}$ that maps transcripts T corresponding to leaves of $\mathcal{T}$ to $\{0,1\}$ which can distinguish between S_0 and S_1 with advantage $1/3$.*[1]

[1]By this, we mean that for all $a \in \{0,1\}$ and $i \in S_a$, $\Pr_{T\sim p_i}[\mathcal{A}(T) = \mathbb{1}[i \in S_0]] \geq 2/3$.

3.B Basic hybrid argument

Here we describe a standard template for showing quantum query complexity lower bounds via a hybrid argument.

Lemma 3.B.1. *Let $\mathcal{E}_0, \mathcal{E}_1$ be quantum channels on n qubits such that for all pure states σ, we have $\|(\mathcal{E}_0 - \mathcal{E}_1)[\sigma]\|_{\mathsf{tr}} \leq \varepsilon$. Let A be any depth-T quantum circuit with access to one of the two channels, and let $s \in \{0,1\}^n$ be the random string output by the circuit. Let p_0, p_1 denote the distribution over s when A has access to $\mathcal{E}_0, \mathcal{E}_1$ respectively. Then $d_{TV}(p_0, p_1) \leq \varepsilon T$.*

Proof. Let $\mathcal{E} = \mathcal{E}_s$ for $s \in \{0,1\}$, and define the channel $\mathcal{U}_i$ which acts by $\mathcal{U}_i(\sigma) = U_i \sigma U_i^\dagger$ where U_i is an associated unitary operator. We proceed via a hybrid argument. The output state of the circuit is given by

$$\sigma^s = \mathcal{U}_T \circ \mathcal{E} \circ \cdots \circ \mathcal{U}_2 \circ \mathcal{E} \circ \mathcal{U}_1[|0^n\rangle\langle 0^n|]$$

for some unitaries $U_1, \ldots, U_T$. For $s' = 1 - s$ and $1 \leq i \leq T$ define

$$\sigma^{(i)} \triangleq \mathcal{U}_T \circ \mathcal{E}_s \circ \cdots \circ \mathcal{U}_{i+1} \circ \mathcal{E}_s \circ \mathcal{U}_i \circ \mathcal{E} \circ \cdots \circ \mathcal{U}_2 \circ \mathcal{E} \circ \mathcal{U}_1[|0^n\rangle\langle 0^n|]\,.$$

Then

$$\begin{aligned}\|\sigma^s - \sigma^{s'}\|_{\mathsf{tr}} &= \left\|\sum_{i=1}^T \sigma^{(i)} - \sigma^{(i-1)}\right\|_{\mathsf{tr}} \leq \sum_{i=1}^T \|\sigma^{(i)} - \sigma^{(i-1)}\|_{\mathsf{tr}} \\ &\leq \sum_{i=1}^T \|(\mathcal{E} - \mathcal{E}_s) \circ \mathcal{U}_{i-1} \circ \mathcal{E} \circ \cdots \circ \mathcal{U}_2 \circ \mathcal{E} \circ \mathcal{U}_1[|0^n\rangle\langle 0^n|]\|_{\mathsf{tr}} \leq T \sup_\sigma \|(\mathcal{E} - \mathcal{E}_s)[\sigma]\|_{\mathsf{tr}},\end{aligned}$$

where the supremum is over all density matrices. By convexity of the trace norm, this bound still holds when the supremum is restricted to pure states σ. By assumption, the above quantity is εT. The total variation distance between p_1 and p_2 as defined in lemma statement is simply the L_1 distance between the diagonals of σ^s and $\sigma^{s'}$, which is upper bounded by $\|\sigma^s - \sigma^{s'}\|_{\mathsf{tr}} \leq \varepsilon T$. □

Supplementary Note 4 – Super-Polynomial Oracle Separations

4.A NISQ vs. BPP

This section is devoted to proving the following theorem.

Theorem 4.A.1 (Restatement of Theorem 2.2)**.** $\mathsf{BPP}^{O_1} \subsetneq \mathsf{NISQ}^{O_1}$ *relative to a classical oracle O_1.*

Our basic strategy is to modify the Simon's oracle into a new classical oracle such that the new oracle is robust to noise. We note that a NISQ algorithm is unable to implement known fault-tolerant quantum computation schemes that can run for any arbitrary quantum circuit with a polynomial number of gates. However, we will still take inspiration from a fault-tolerant quantum computation scheme [24] to define a certain "robustified Simon's oracle" relative to which we obtain a super-polynomial separation between BPP and NISQ. As we will show, because the fault-tolerant scheme of [24] is robust not just to local depolarizing noise but to arbitrary local noise occurring with sufficiently small constant rate, the NISQ algorithm that we give will ultimately be robust under this stronger noise model as well (see Remark 4.A.1).

4.A.1 Recursively-defined concatenated code

We consider a Calderbank-Shor-Steane (CSS) code built from two classical linear codes C_1, C_2, where $C_1 \triangleq C$ is a punctured doubly-even self-dual code and $C_2 \triangleq C^\perp$ (we refer the reader to [24] for background on these notions). We consider C_1, C_2 to be over m classical bits. The corresponding CSS code encodes a single logical qubit into m physical qubits. Let $\mathbf{1}_m$ denote the all-ones vector of length m (when the subscript is clear from context, we will omit it). The two code words in the CSS code are given by

$$|S_0\rangle = \frac{1}{\sqrt{|C^\perp|}} \sum_{w \in C^\perp} |w\rangle\,, \quad |S_1\rangle = \frac{1}{\sqrt{|C^\perp|}} \sum_{w \in C^\perp} |w \oplus \mathbf{1}_m\rangle\,, \tag{2}$$

where $\oplus$ denotes addition over $\mathbb{Z}_2^m$ (i.e., it is the bit-wise XOR). Denote by d the number of errors that can be corrected by the CSS code. The two parameters m and d are both considered to be constant. We define

$$A_0 \triangleq \left\{ w \oplus x \,\middle|\, w \in C^\perp, x \in \{0,1\}^m, |x| \le d \right\} \tag{3}$$

$$A_1 \triangleq \left\{ w \oplus x \,\middle|\, w \in C^\perp \oplus \mathbf{1}, x \in \{0,1\}^m, |x| \le d \right\}, \tag{4}$$

where $C^\perp \oplus \mathbf{1}$ denotes the set $\{x \oplus \mathbf{1} \,|\, x \in C^\perp\}$ and $|x|$ is the number of 1's in x.

Lemma 4.A.1 (Disjointness of A_0 and A_1)**.** *With the above definitions, we have*

$$A_0 \cap A_1 = \emptyset. \tag{5}$$

Proof. This lemma follows from the definition of d. Assume that the intersection is non-empty, i.e., $A_0 \cap A_1 \neq \emptyset$. Hence $w_1 \oplus x_1 = w_2 \oplus \mathbf{1} \oplus x_2$ for some $w_1, w_2 \in C^\perp$, $x_1, x_2 \in \{0,1\}^m$ with $|x_1|, |x_2| \le d$. Let us define $E_1 = \bigotimes_{i=1}^m X^{x_{1i}}$, $E_2 = \bigotimes_{i=1}^m X^{x_{2i}}$ for single-qubit Pauli X. Then,

$$\begin{aligned} E_1 |S_0\rangle &= \frac{1}{\sqrt{|C^\perp|}} \sum_{w \in C^\perp} |w \oplus x_1\rangle = \frac{1}{\sqrt{|C^\perp|}} \sum_{w \in C^\perp} |w \oplus w_1 \oplus x_1\rangle \\ &= \frac{1}{\sqrt{|C^\perp|}} \sum_{w \in C^\perp} |w \oplus w_2 \oplus x_2 \oplus \mathbf{1}\rangle = \frac{1}{\sqrt{|C^\perp|}} \sum_{w \in C^\perp} |w \oplus \mathbf{1} \oplus x_2\rangle = E_2 |S_1\rangle\,. \end{aligned}$$

Hence $\langle S_1| E_2 E_1 |S_0\rangle = 1$, where E_1 and E_2 are Pauli operators with weight at most d. This contradicts the definition of d. □

We now recall the recursive concatentation construction from [24]. Given an integer $r > 0$, we define the following code va $r+1$ levels of recursion. Each level encodes a single qubit using the above CSS code over m qubits from the previous level. Hence, a single qubit in the top level is encoded by a total of m^r qubits in the bottom level. More formally, for each r, we will define two sets $B_0^{(r)}, B_1^{(r)}$ over m^r-bit strings as follows. These sets contain the computational basis states that are used to span the recursively-defined concatenated code.

Definition 4.A.1 (Basis of the concatenated code)**.** *For $r = 1$, $B_0^{(1)} \triangleq C^\perp$ and $B_1^{(1)} \triangleq C^\perp \oplus \mathbf{1}$. For $r > 1$, we define $B_0^{(r)}, B_1^{(r)}$ recursively,*

$$B_0^{(r)} \triangleq \left\{ (v_1, \ldots, v_m) \in \{0,1\}^{m^r} \,\middle|\, w \in C^\perp, v_i \in B_{w_i}^{(r-1)}, \forall i = 1, \ldots, m \right\},$$

$$B_1^{(r)} \triangleq \left\{ (v_1, \ldots, v_m) \in \{0,1\}^{m^r} \,\middle|\, w \in C^\perp \oplus \mathbf{1}, v_i \in B_{w_i}^{(r-1)}, \forall i = 1, \ldots, m \right\}.$$

The two code words in the recursively-defined concatenated code are then given by

$$|R_b\rangle = \frac{1}{\sqrt{|B_b^{(r)}|}} \sum_{x \in B_b^{(r)}} |x\rangle, \quad b \in \{0,1\}. \tag{6}$$

For each r, we also define two sets $A_0^{(r)}, A_1^{(r)}$ over m^r-bit strings that correspond to the neighborhoods around $B_0^{(r)}, B_1^{(r)}$ induced by errors.

Definition 4.A.2 (Neighborhood of $B_0^{(r)}, B_1^{(r)}$)**.** *For $r = 1$, $A_0^{(1)} \triangleq A_0$ and $A_1^{(1)} \triangleq A_1$. By Eq. (5), $A_0^{(r)} \cap A_1^{(r)} = \emptyset$. For $r > 1$, we define $A_0^{(r)}, A_1^{(r)}$ recursively,*

$$A_0^{(r)} \triangleq \left\{ (v_1, \ldots, v_m) \in \{0,1\}^{m^r} \,\middle|\, w_0 \in C^\perp, x_0 \in \{0,1\}^m, |x_0| \le d, v_i \in A_{w_{0i}}^{(r-1)} \ \forall i \text{ s.t. } x_{0i} = 0 \right\},$$
$$A_1^{(r)} \triangleq \left\{ (v_1, \ldots, v_m) \in \{0,1\}^{m^r} \,\middle|\, w_1 \in C^\perp \oplus \mathbf{1}, x_1 \in \{0,1\}^m, |x_1| \le d, v_i \in A_{w_{1i}}^{(r-1)} \ \forall i \text{ s.t. } x_{1i} = 0 \right\}.$$

We can prove the following two lemmas.

Lemma 4.A.2 (Structure of $A_0^{(r)}$ and $A_1^{(r)}$)**.** *For all $r \ge 1$, we have*

$$A_0^{(r)} \oplus \mathbf{1} = A_1^{(r)}.$$

Proof. We consider a proof by induction on $r \ge 1$. By definition of A_0 and A_1, we have $A_0^{(1)} \oplus \mathbf{1} = A_1^{(1)}$, which establishes the base case of $r = 1$. For $r > 1$, we show that for any $(v_1, \ldots, v_m) \in A_0^{(r)}$, we have $(v_1, \ldots, v_m) \oplus \mathbf{1} \in A_1^{(r)}$. Consider w_0, x_0 corresponding to $(v_1, \ldots, v_m)$. Using $v_i \in A_{w_{0i}}^{(r-1)}$ for all i with $x_{0i} = 0$ and the inductive hypothesis that $A_0^{(r-1)} \oplus \mathbf{1} = A_1^{(r-1)}$, we have $v_i \oplus \mathbf{1} \in A_{w_{0i} \oplus 1}^{(r-1)}$ for all i with $x_{0i} = 0$. Hence, by considering $w_1 = w_0 \oplus \mathbf{1}$ and $x_1 = x_0$, we have $(v_1, \ldots, v_m) \oplus \mathbf{1} \in A_1^{(r)}$. Similarly, we can show that for any $(v_1, \ldots, v_m) \in A_1^{(r)}$, we have $(v_1, \ldots, v_m) \oplus \mathbf{1} \in A_0^{(r)}$. Therefore, we have shown that $A_0^{(r)} \oplus \mathbf{1} = A_1^{(r)}$. □

Lemma 4.A.3 (Disjointness of $A_0^{(r)}$ and $A_1^{(r)}$)**.** *For all $r \ge 1$, we have*

$$A_0^{(r)} \cap A_1^{(r)} = \emptyset.$$

Proof. The base case is given in Lemma 4.A.1. Now for the induction step, assume $A_0^{(r-1)} \cap A_1^{(r-1)} = \emptyset$. Eq. (5) implies that the minimum Hamming distance between any two bitstrings in $C^\perp$ and $C^\perp \oplus \mathbf{1}$ is at least $2d+1$. Hence, for any $w_1 \in C^\perp$ and $w_2 \in C^\perp \oplus \mathbf{1}$, after removing at most $2d$ bits (the bits with $x_{1i} = 1$ or $x_{2i} = 1$), there still exists an index i among the rest of the bits (i.e., the bits with $x_{1i} = 0$ and $x_{2i} = 0$) such that $w_{1i} \ne w_{2i}$. Because $A_{w_{1i}}^{(r-1)} \cap A_{w_{2i}}^{(r-1)} = \emptyset$ by the induction hypothesis, we have $A_0^{(r)} \cap A_1^{(r)} = \emptyset$. □

4.A.2 Robustified Simon's problem

Given a large enough integer n, we consider Simon's problem over $n' = 2^{\Theta(\log(n)^c)}$ bits for a constant $0 < c < 1$. Here $1/c$ corresponds to the constant c_2 from Theorem 10 of [24]. We consider $r = \Theta(\log\log(n'))$ and encode each of the n' bits using m^r bits. Because $m = \mathcal{O}(1)$, we have $m^r n' = 2^{\Theta(\log(n)^c)} < n$ for large enough n.

Given a classical function $f_s : \{0,1\}^{n'} \to \{0,1\}^{n'}$ from Simon's problem with secret string $s \in \{0,1\}^{n'}$, we define a classical function $\widetilde{f}_s : \{0,1\}^n \to \{0,1\}^{m^r n'}$ as follows. Let x be an n-bit string. We focus on the first $m^r n'$ bits of x and divide them into n' m^r-bit strings as $x_1, \ldots, x_{n'}$. We first define $\widetilde{f}^0_s : \{0,1\}^n \to \{0,1\}^{n'}$ as follows,

$$\widetilde{f}^0_s(x) \triangleq \begin{cases} f_s(b_1 \ldots b_{n'}), & \text{if } \exists!\, b_1, \ldots, b_{n'} \in \{0,1\}, \text{ s.t. } x_i \in A^{(r)}_{b_i}, \forall i = 1, \ldots, n', \\ 0^{n'}, & \text{otherwise} \end{cases}. \tag{7}$$

We use $\exists!$ to denote "there exists a unique choice". Because $A^{(r)}_0$ and $A^{(r)}_1$ are disjoint, there either exists a unique choice of $b_1, \ldots, b_{n'}$ or does not exist any choice of $b_1, \ldots, b_{n'}$ that satisfies $x_i \in A^{(r)}_{b_i}, \forall i = 1, \ldots, n'$. Letting $[\widetilde{f}^0_s(x)]_k$ denote the kth bit of $\widetilde{f}^0_s(x)$, we define the function $\widetilde{f}_s : \{0,1\}^n \to \{0,1\}^{m^r n'}$ by

$$\widetilde{f}_s(x) \triangleq \left(\underbrace{[\widetilde{f}^0_s(x)]_1, \ldots, [\widetilde{f}^0_s(x)]_1}_{m^r \text{ times}}, \ldots, \underbrace{[\widetilde{f}^0_s(x)]_{n'}, \ldots, [\widetilde{f}^0_s(x)]_{n'}}_{m^r \text{ times}} \right) \in \{0,1\}^{m^r n'}. \tag{8}$$

The function $\widetilde{f}_s$ can be considered as the robust version of f_s, where the output bitstring is stable over a large number of bitstrings.

Let $U_{\widetilde{f}_s}$ be the unitary from Eq. (9).

$$U_{\widetilde{f}_s} |x\rangle |y\rangle = |x\rangle |y \oplus \widetilde{f}_s(x)\rangle, \quad \forall x \in \{0,1\}^n, y \in \{0,1\}^{m^r n'}. \tag{9}$$

We denote by $O_{\widetilde{f}_s}$ the oracle which applies this unitary. Then we have the following theorem, which is the main result of this section and implies Theorem 2.2:

Theorem 4.A.2. *For λ sufficiently small, there is a NISQ_λ algorithm which, given oracle access to $O_{\widetilde{f}_s}$, can determine whether f_s is 2-to-1 or 1-to-1 with constant advantage in time at most $\mathcal{O}(\text{poly}(n))$. By contrast, any classical algorithm with access to $O_{\widetilde{f}_s}$ requires at least $\Omega(\text{superpoly}(n))$ time, to determine whether f_s is 2-to-1 or 1-to-1 with constant advantage. Thus, relative to oracles O of this form, $\mathsf{BPP}^O \subsetneq \mathsf{NISQ}^O$.*

Our strategy for proving this theorem is to combine the following two ingredients. First, we draw on tools underlying the error correction scheme in Theorem 10 of [24]. This scheme requires the algorithm to be able to initialize constant-error noisy zero states in the middle of the circuit, which our computational model does not allow for. Our second proof ingredient is then to leverage the noisy majority vote approach in Theorem 4 of [22] for distilling constant-error noisy zero states in the middle of the circuit from constant-error noisy zero states prepared at the beginning of the circuit. Putting these together, we can utilize a noisy quantum machine with $n' \log(n')^{c_1} \times 2^{\Theta(\log(n')^c)} = \text{poly}(n)$ qubits (and a similar number of gates) and noise rate $\lambda < \lambda_0$ (for a small constant λ_0) to run an encoded version of Simon's algorithm on the oracle $O_{\widetilde{f}_s}$. We will find that our classical oracle, which essentially implements a classical error correction code, interfaces nicely with the quantum error correction scheme of [24], enabling our algorithm to work.

4.A.3 Simulating fault-tolerant Simon's in NISQ with exponential overhead

Recall that Simon's algorithm consists of three steps: (i) prepare the all-plus state on the first half of the qubits and the all-zero state on the second half of the qubits, (ii) query the oracle, and (iii) apply the Hadamards to the first half of the qubits followed by measuring them in the

computational basis. In this section, we describe how to implement encoded versions of the first and last steps. The main guarantees for these steps are respectively given by Lemmas 4.A.4-4.A.5 and Lemma 4.A.6 below.

To set up the proof, we require some definitions from [24] so that we can state the needed theorems from [24] and [22]. Accordingly, let us recall the following notions of (r,k)-sparse sets and (r,k)-deviated states from [24].

Definition 4.A.3 ((r,k)-sparse set)**.** *An (r,k)-sparse set of qubits over many blocks of the m^r qubits is defined recursively as follows. A set A of qubits over many blocks of m qubits is $(1,k)$-sparse if and only if every block has at most k qubits that are in A. A set A of qubits over many blocks of m^r qubits is (r,k)-sparse if and only if for every block, by treating the m^r qubits as m sub-blocks of m^{r-1} qubits, there are at most k sub-blocks that are not $(r-1,k)$-sparse.*

Definition 4.A.4 ((r,k)-deviate)**.** *A state ρ is said to be (r,k)-deviated from ρ' if k is the minimum integer such that there exists an (r,k)-sparse set of qubits A, such that $\rho_{A^c} = \rho'_{A^c}$. Here, we denote ρ_{A^c} to be the reduced density matrix of ρ on the qubits not in set A.*

We will also need the definitions of location and quantum computation code from [24].

Definition 4.A.5 (Location)**.** *A set $(q_1,\ldots,q_a,t)$ is a* location *in a quantum circuit Q if the qubits $q_1,\ldots,q_a$ participated in the same gate in Q at layer t, and no other qubit participated in that gate. If a qubit q did not participate in any gate at layer t, then (q,t) is a location in Q as well.*

Definition 4.A.6 (Quantum computation code)**.** *A quantum code C encoding a single logical qubit into m physical qubits is called a* quantum computation code *if it is accompanied with a universal set of gates $\mathcal{G}$ with fault tolerant procedures, including fault tolerant encoding, decoding, and error correction procedures. Moreover, we require that* (i) *all procedures use only gates from $\mathcal{G}$, and* (ii) *the error correction procedure takes any m-qubit density matrix to a state in the code space.*

Apart from state encoding and decoding, the quantum computation code C also encodes any gate $g \in \mathcal{G}$ into a circuit $P(g)$ such that for any pure state $|\psi\rangle$, $P(g)$ maps the state encoding of ψ under C to the state encoding of $g|\psi\rangle$. If g is a k-qubit gate, then the circuit $P(g)$ acts on k blocks of m qubits (each block encoding one logical qubit) possibly with other ancilla qubits.

We are now prepared to recall the threshold theorem of [24], which we state in the context of probabilistic qubit-wise noise (with probability λ, an arbitrary single-qubit quantum channel is applied on the qubit). In the rest of the section, we consider any failure probability $\delta > 0$, and a (noiseless) quantum circuit Q with $2n'$ input qubits, depth t, and v locations. Let C be a quantum computation code with gates $\mathcal{G}$ that corrects d errors. Let

$$V : \mathbb{C}^2 \to (\mathbb{C}^2)^{\otimes m^r}$$

be the encoding map for the code given by recursively concatenating C a total of $r = \mathcal{O}(\log\log(v/\delta))$ times. Using key lemmas for establishing the threshold theorem from [24] (Theorem 10 therein), we can extract the following two results:

Theorem 4.A.3 (Lemma 8 and 10 from [24])**.** *There is an absolute constant $\lambda_c \in [0,1]$ such that for any $\lambda < \lambda_c$, there exists a λ-noisy quantum circuit Q' which can initialize ancillary qubits at any time (these ancillary qubits are also subject to qubit-wise noise of λ) during the computation and satisfies the following. Q' operates on $m^r n'$ qubits and has depth $\mathcal{O}(t\,\mathrm{polylog}(v/\delta))$, and the output state ρ of Q' is (r,d)-deviated from*

$$V^{\otimes n'} Q |0^{n'}\rangle\langle 0^{n'}| Q^\dagger (V^{\otimes n'})^\dagger$$

with probability $1-\delta$ over the local noise.

Theorem 4.A.4 (Lemma 8, 9, and 10 from [24]). *There is an absolute constant $\lambda_c \in [0,1]$ such that for any $\lambda < \lambda_c$, there exists a λ-noisy quantum circuit Q' which can initialize ancillary qubits at any time (these ancillary qubits are also subject to qubit-wise noise of λ) during the computation, and a classical postprocessing algorithm $\mathcal{A}$ based on recursive majority vote, that satisfies the following. Q' operates on $m^r n'$ qubits and has depth $\mathcal{O}(t\,\mathrm{polylog}(v/\delta))$. Let σ be any n'-qubit state. Let $\mathcal{D}$ be the n'-bit string distribution generated by measuring $Q\sigma Q^\dagger$ in the computational basis. For any state ρ that is (r,d)-deviated from*

$$V^{\otimes n'}\sigma(V^{\otimes n'})^\dagger,$$

applying a λ-noisy computational basis measurements on the output state of Q' given input state ρ, followed by the classical algorithm $\mathcal{A}$, produces a distribution $\mathcal{D}'$ equal to $\mathcal{D}$ with probability $1-\delta$ over the local noise.

Our CSS code defined at the beginning of Subsection 4.A.1 in Eqs. (2), (3), (4) and the surrounding text, which corrects d errors is a suitable quantum computational code for the purposes of the above Theorem. Indeed, the construction in the proof of Theorem 4.A.3 and 4.A.4 is to build the concatenated code described in Definition 4.A.1 and Eq. (6) and the surrounding text, and then to show that it has suitable fault-tolerant quantum error correction properties.

As mentioned above, the key difference between our model for noisy quantum circuits versus the one considered in that work is that the latter allows the circuit to initialize ancillary qubits at any point in the computation, whereas in our setting all qubits are present at time zero. In [22] it was shown how to pass from the latter setting to the former with a blowup in total number of qubits that is exponential in the depth of the original circuit.

Theorem 4.A.5 (Theorem 4 from [22]). *There exists an absolute constant $\lambda_c \in [0,1]$ such that for any non-negative $\lambda < \lambda_c$ and any $t \in \mathbb{N}$, there exists a λ-noisy quantum circuit of depth t operating on 3^t qubits initialized to the all-zero state such that the output state's first qubit is in the state $|0\rangle$ with probability at least $1-\lambda$.*

We now use Theorems 4.A.3, 4.A.4, and 4.A.5 to argue in Lemma 4.A.4 (resp. Lemma 4.A.5) that we can prepare an encoding of the all-plus state (resp. all-zero state) over n' qubits using $\mathrm{poly}(n)$ total qubits including ancillas, thus realizing an encoded version of the first step in Simon's algorithm.

Lemma 4.A.4. *Suppose $n' \leq \exp(\log^c n)$ for $0 < c < 1$ a sufficiently small constant. There exists an absolute constant $\lambda_c \in [0,1]$ such that for any non-negative $\lambda < \lambda_c$, there exists a λ-noisy quantum circuit which operates on n' input qubits and $\mathrm{poly}(n)$ ancillary qubits and has $\mathrm{polylog}(n)$ layers, such that with probability at least $1-\mathcal{O}(1/n')$ over the local noise, the output state is $(r, d/2)$-deviated from the state*

$$V^{\otimes n'}H^{\otimes n'}|0^{n'}\rangle\langle 0^{n'}|H^{\otimes n'}(V^{\otimes n'})^\dagger \tag{10}$$

for $r = \log\log(n')$.

Proof. By Theorem 4.A.3, there is a λ-noisy circuit with $\mathrm{polylog}(n')$ layers and $n'\mathrm{polylog}(n')$ qubits, which can initialize qubits at any time, such that with probability at least $1-\mathcal{O}(1/n')$ over the local noise, the output state is $(r, d/2)$-deviated from the state in Eq. (10). By Theorem 4.A.5, for each qubit that is initialized at some arbitrary time $t \leq \mathrm{polylog}(n')$, we can append to Q' a λ-noisy circuit of depth t operating on 3^t qubits initialized at time zero, such that the first qubit of the output state of this circuit can play the role of the qubit initialized at time t in Q'. Altogether, we obtain a circuit whose depth is the same as Q' but which now operates on at most $3^{\mathrm{polylog}(n')} \cdot n'\mathrm{polylog}(n')$

qubits. This is at most $\text{poly}(n)$ provided that the constant c in the in the Lemma is sufficiently small. $\square$

Using the same proof as the above lemma, we can establish the following.

Lemma 4.A.5. *Suppose $n' \leq \exp(\log^c n)$ for $0 < c < 1$ a sufficiently small constant. There exists an absolute constant $\lambda_c \in [0,1]$ such that for any non-negative $\lambda < \lambda_c$, there exists a λ-noisy quantum circuit which operates on n' input qubits and $\text{poly}(n)$ ancillary qubits and has $\text{polylog}(n)$ layers, such that with probability at least $1 - \mathcal{O}(1/n')$ over the local noise, the output state is $(r, d/2)$-deviated from the state*

$$V^{\otimes n'}|0^{n'}\rangle\langle 0^{n'}|(V^{\otimes n'})^\dagger$$

for $r = \log\log(n')$.

Next, we use Theorems 4.A.3 and 4.A.5 to argue that we can take any state on $m^r n'$ qubits which is not too deviated from a codeword, noisily apply Hadamard transform, and apply fault-tolerant measurement to the result. This realizes an encoded version of the last step of Simon's algorithm. The noisy quantum circuit for this uses $\text{poly}(n)$ total qubits including ancillas.

Lemma 4.A.6. *Suppose $n' \leq \exp(\log^c n)$ for $0 < c < 1$ a sufficiently small constant and for $r = \text{polylog}(n')$. There exists an absolute constant $\lambda_c \in [0,1]$ such that for any non-negative $\lambda < \lambda_c$, there exists a λ-noisy quantum circuit Q' which operates on $m^r n'$ input qubits and $\text{poly}(n)$ ancillary qubits and has $\text{polylog}(n')$ layers, such that the following holds. Let $\mathcal{A}$ be the classical post-processing procedure based on recursive majority vote from Theorem 4.A.4.*

For k satisfying $k \leq d$, let input state ρ be (r,k)-deviated from the state

$$V^{\otimes n'}\sigma(V^{\otimes n'})^\dagger.$$

Let $\mathcal{D}$ be the classical distribution over $\{0,1\}^{n'}$ generated by measuring

$$H^{\otimes n'}\sigma H^{\otimes n'}$$

in the computational basis. Then if one applies Q' to ρ, noisily measures the output state under the computational basis, and applies $\mathcal{A}$ to the classical outcome, then the resulting distribution over $\{0,1\}^{n'}$ is identical to $\mathcal{D}$ with probability at least $1 - \mathcal{O}(1/n')$ over the local noise.

Proof. Because ρ is (r,k)-deviated from $V^{\otimes n'}\sigma(V^{\otimes n'})^\dagger$ for $k \leq d$, we can apply Theorem 4.A.4 to obtain a λ-noisy quantum circuit Q'' operating on $m^r n'$ qubits with $\text{polylog}(n')$ layers which satisfies the desiderata of the lemma. The caveat is that the circuit has to be able to initialize ancilla qubits at any time.

We can address this similarly to the proof of Lemma 4.A.4. By Theorem 4.A.5, for each qubit that is initialized at some arbitrary time $t \leq \text{polylog}(n')$, we can append to Q'' a λ-noisy circuit of depth t operating on 3^t qubits initialized at time zero, such that the first qubit of the output state of this circuit can play the role of the qubit initialized at time t in Q''. Altogether, we obtain a circuit Q' whose depth is the same as Q'' but which now operates on at most $3^{\text{polylog}(n')} \cdot n'\text{polylog}(n') \leq \text{poly}(n)$ qubits. $\square$

4.A.4 Stability against deviation in the robustified Simon's oracle

So far, Lemma 4.A.4 implies that we can realize the first step of Simon's in an encoded fashion: noisily prepare a state, call it ρ_1, which is only slightly deviated from an encoding of the all-plus

state. And Lemma 4.A.6 implies that given a state, call it ρ_2, which is only slightly deviated from the output of the robustified Simon's oracle, we can simulate the last step of Simon's algorithm in the presence of noise. In order to apply Lemma 4.A.6 however, it remains to verify that when we go from ρ_1 to ρ_2 by invoking the robustified oracle in the second step of Simon's, the sparsity of the deviations in ρ_1 is preserved. We show this in Lemma 4.A.7 below.

First, recall the unitary $U_{\widetilde{f}_s}$ from Eq. (9). Note that by construction, $\widetilde{f}_s$ only depends non-trivially on the first $m^r n' < n$ bits of its input. By defining $\widetilde{f}_s^* : \{0,1\}^{m^r n'} \to \{0,1\}^{m^r n'}$ to be the function $\widetilde{f}_s$ restricted to the first $m^r n'$ bits, we can rewrite (9) as

$$U_{\widetilde{f}_s} |x_1\rangle |x_2\rangle |y\rangle = |x_1\rangle |x_2\rangle |y \oplus \widetilde{f}_s^*(x_1)\rangle , \quad \forall x_1 \in \{0,1\}^{m^r n'}, \, x_2 \in \{0,1\}^{n - m^r n'}, \, y \in \{0,1\}^{m^r n'}.$$

We see from the above equation that $U_{\widetilde{f}_s}$ acts trivially on the $|x_2\rangle$ part of the input state. So let us define $U_{\widetilde{f}_s^*}$ as the restriction of $U_{\widetilde{f}_s}$ to its $|x_1\rangle$ and $|y\rangle$, subsystems, namely

$$U_{\widetilde{f}_s^*} |x_1\rangle |y\rangle = |x_1\rangle |y \oplus \widetilde{f}_s^*(x_1)\rangle , \quad \forall x_1 \in \{0,1\}^{m^r n'}, \, y \in \{0,1\}^{m^r n'}.$$

With the above notations for the oracle, we can now prove the following lemma showing the classical function $\widetilde{f}_s^*$ preserves the deviation metric. We note that, on the other hand, the ordinary Simon's function f_s does not have the same property.

Lemma 4.A.7 (Stability of the robustified classical oracle)**.** *Consider a Hilbert space $\mathcal{H}$ which decomposes into subsystems as $\mathcal{H} \simeq \mathcal{H}_{\text{main},1} \otimes \mathcal{H}_{\text{anc},1}$ where $\mathcal{H}_{\text{main},1} \simeq (\mathbb{C}^2)^{\otimes(m^r n')}$ and $\mathcal{H}_{\text{anc},1} \simeq (\mathbb{C}^2)^{\otimes(m^r n')}$. Further let $\rho^0 = V^{\otimes n'} H^{\otimes n'} |0^{n'}\rangle\langle 0^{n'}| H^{\otimes n'} (V^{\otimes n'})^\dagger$ and $\sigma^0 = V^{\otimes n'} |0^{n'}\rangle\langle 0^{n'}| (V^{\otimes n'})^\dagger$.*

Given any $k \leq d$, if $\rho \otimes \sigma$ is (r,k)-deviated from $\rho^0 \otimes \sigma^0$, then $\operatorname{tr}_{\mathcal{H}_{\text{anc},1}}\Big\{U_{\widetilde{f}_s^*}(\rho \otimes \sigma) U_{\widetilde{f}_s^*}^\dagger\Big\}$ is (r,k)-deviated from $\operatorname{tr}_{\mathcal{H}_{\text{anc},1}}\Big\{U_{\widetilde{f}_s^*}(\rho^0 \otimes \sigma^0) U_{\widetilde{f}_s^*}^\dagger\Big\}$.

Proof. Consider the set $S \subset \{0,1\}^{m^r n'}$ defined by

$$S \triangleq \{t \in \{0,1\}^{m^r n'} \,\big|\, t = q_1 \cdots q_{n'} \, , \, q_i \in B_0^{(r)} \cup B_1^{(r)}\} \, .$$

Moreover, if $A = \{a_1, a_2, ..., a_{|A|}\}$ is a subset of $\{1, 2, ..., m^r n'\}$ where $a_1 < a_2 < \cdots < a_{|A|}$, then further define

$$S_A \triangleq \{u \in \{0,1\}^{|A|} \,\big|\, u = t|_A \text{ for some } t \in S\} \, .$$

Here $t|_A$ means the restriction of t to its bits in locations $a_1, a_2, ..., a_{|A|}$.

Because ρ is (r,k)-deviated from ρ^0, there exists an (r,k)-sparse subset A of qubits, such that $\rho_{A^c} = \rho^0_{A^c}$. Decomposing the Hilbert space $\mathcal{H}_{\text{main},1}$ as $\mathcal{H}_{\text{main},1} \simeq \mathcal{H}_A \otimes \mathcal{H}_{A^c}$, then using our notations above we can write

$$\rho^0_{A^c} = \rho_{A^c} = \sum_{t,t' \in S_{A^c}} c_{t,t'} |t\rangle\langle t'|$$

for some constants $c_{t,t'} \in \mathbb{C}$. We further know, by hypothesis, that we can write

$$\rho^0 = \sum_{t,t' \in S_{A^c}} O^A_{t,t'} \otimes |t\rangle\langle t'|$$

for some operators $O^A_{t,t'}$ such that $\operatorname{tr}(O^{A^c}_{t,t'}) = c_{t,t'}$. If all we know about ρ is that it is (r,k)-deviated from ρ^0, then *a priori* we only have the more general decomposition

$$\rho = \sum_{t,t' \in S_{A^c}} \widetilde{O}^A_{t,t'} \otimes |t\rangle\langle t'| + \sum_{\substack{t,t' \in \{0,1\}^{|A^c|} \\ \text{one of } t,t' \text{ is not in } S_{A^c}}} Q^A_{t,t'} \otimes |t\rangle\langle t'| \, ,$$

where $\mathrm{tr}(\widetilde{O}^A_{t,t'}) = c_{t,t'}$ such that $t, t' \in S_{A^c}$, and $\mathrm{tr}(Q^A_{t,t'}) = 0$ for all $t, t' \in \{0,1\}^{|A^c|}$ such that one of t, t' is not in S_{A^c}. But observe that because

$$\sum_{\substack{t,t' \in \{0,1\}^{|A^c|} \\ t,t' \notin S_{A^c}}} Q^A_{t,t'} \otimes |t\rangle\langle t'|$$

has trace zero and is positive semi-definite (as it is obtained by left- and right-multiplying ρ by the projector $\sum_{t \notin S_{A^c}} |t\rangle\langle t|$), we must have $Q^A_{t,t'} = 0$ for all $t, t' \notin S_{A^c}$. On the other hand, if $|1\rangle, \ldots, ||A|\rangle$ is an orthonormal basis for $\mathcal{H}_A$, then the fact that the 2×2 principal minors of ρ must be nonnegative implies that for any $1 \le i, j \le |A|$ and any $t \in S_{A^c}$, $t' \notin S_{A^c}$ we have

$$(\langle i| \otimes \langle t|)\rho(|i\rangle \otimes |t\rangle) \cdot (\langle j| \otimes \langle t'|)\rho(|j\rangle \otimes |t'\rangle) \ge (\langle i| \otimes \langle t|)\rho(|j\rangle \otimes |t'\rangle) \cdot (\langle j| \otimes \langle t'|)\rho(|i\rangle \otimes |t\rangle)\,.$$

Because we have already shown that $Q^A_{t',t'} = 0$, the left-hand side is zero. On the other hand, because ρ is Hermitian, the right-hand side is nonnegative. The right-hand side is the squared magnitude of $(Q^A_{t,t'})_{ij}$, so we conclude that $Q^A_{t,t'} = 0$ for all t, t' for which at least one of t, t' is not in S_{A^c}. In other words, ρ actually has the decomposition

$$\rho = \sum_{t,t' \in S_{A^c}} \widetilde{O}^A_{t,t'} \otimes |t\rangle\langle t'|\,.$$

From the definition of σ^0, we have

$$\sigma^0 = \sum_{y,y' \in (B_0^{(r)})^{n'}} \sigma^0_{y,y'} |y\rangle\langle y'|\,.$$

We can then use the fact that σ is (r,k)-deviated from σ^0 to show that

$$\sigma = \sum_{y,y' \in (A_0^{(r)})^{n'}} \sigma_{y,y'} |y\rangle\langle y'|\,.$$

This is because the definition of $A_0^{(r)}$ ensures that it contains all bitstrings that can be generated by taking any bitstring in $B_0^{(r)}$ and flipping the bits in an (r,d)-sparse set of bits. We are now ready to study the effect under the oracle $U_{\tilde{f}_s^*}$.

Writing $\widetilde{O}^A_{t,t'} = \sum_{u,u' \in \{0,1\}^{|A|}} \widetilde{b}_{t,t',u,u'} |u\rangle\langle u'|$, we then have

$$U_{\tilde{f}_s^*}(\rho \otimes \sigma) U^\dagger_{\tilde{f}_s^*} = \sum_{\substack{u,u' \in \{0,1\}^{|A|} \\ t,t' \in S_{A^c} \\ y,y' \in (A_0^{(r)})^{n'}}} \widetilde{b}_{t,t',u,u'} \sigma_{y,y'} |u\rangle\langle u'| \otimes |t\rangle\langle t'| \otimes |y \oplus \widetilde{f}_s^*(ut)\rangle\langle y' \oplus \widetilde{f}_s^*(u't')|\,. \tag{11}$$

Recall that A is (r,k)-sparse. Since $k \le d$, on account of (7), (8), and the definition of (r,k)-sparseness, we have that $\widetilde{f}_s^*(ut)$ only depends on t (and likewise $\widetilde{f}_s^*(u't')$ only depends on t'), and so in a slight abuse of notation we write $\widetilde{f}_s^*(ut) = \widetilde{f}_s^*(t)$ so that (11) becomes

$$U_{\tilde{f}_s^*}(\rho \otimes \sigma) U^\dagger_{\tilde{f}_s^*} = \sum_{\substack{u,u' \in \{0,1\}^{|A|} \\ t,t' \in S_{A^c} \\ y,y' \in (A_0^{(r)})^{n'}}} \widetilde{b}_{t,t',u,u'} \sigma_{y,y'} |u\rangle\langle u'| \otimes |t\rangle\langle t'| \otimes |y \oplus \widetilde{f}_s^*(t)\rangle\langle y' \oplus \widetilde{f}_s^*(t')|\,.$$

We similarly write $\widetilde{f}_s^0(ut) = \widetilde{f}_s^0(t)$, and define $a_{t,t'} \triangleq \langle \widetilde{f}_s^0(t') | \widetilde{f}_s^0(t)\rangle$. Consider the following two cases that cover all possibilities of $a_{t,t'}$.

1. $a_{t,t'} = 0$: In this case, $\widetilde{f}_s^0(t')$ and $\widetilde{f}_s^0(t)$ have at least one bit which differs. Say their j-th bits differ, i.e. $[\widetilde{f}_s^0(t)]_j \neq [\widetilde{f}_s^0(t')]_j$. Let $[y]_{i,...,j}$ for $i \leq j$ denote the bitstring $(y_i, y_{i+1}, ..., y_j)$, with similar notation for y'. For the two bitstrings $[y]_{jm^r+1,...,(j+1)m^r}, [y']_{jm^r+1,...,(j+1)m^r} \in A_0^{(r)}$, Lemma 4.A.2 gives

$$[y]_{jm^r+1,...,(j+1)m^r} \oplus (\underbrace{[\widetilde{f}_s^0(t)]_j, ..., [\widetilde{f}_s^0(t)]_j}_{m^r \text{ times}}) \in A^{(r)}_{\widetilde{f}_s^0(t)_j}$$
$$[y']_{jm^r+1,...,(j+1)m^r} \oplus (\underbrace{[\widetilde{f}_s^0(t')]_j, ..., [\widetilde{f}_s^0(t')]_j}_{m^r \text{ times}}) \in A^{(r)}_{\widetilde{f}_s^0(t')_j}.$$

Using Lemma 4.A.3 on the disjointness of $A_0^{(r)}$ and $A_1^{(r)}$, we have

$$[y \oplus \widetilde{f}_s^*(t)]_{jm^r+1,...,(j+1)m^r} \neq [y' \oplus \widetilde{f}_s^*(t')]_{jm^r+1,...,(j+1)m^r}\,.$$

Thus, we have $\langle y' \oplus \widetilde{f}_s^*(t') | y \oplus \widetilde{f}_s^*(t)\rangle = 0$.

2. $a_{t,t'} = 1$: All bits in $\widetilde{f}_s^0(t'), \widetilde{f}_s^0(t)$ are the same. Hence, $\langle y' \oplus \widetilde{f}_s^*(t') | y \oplus \widetilde{f}_s^*(t)\rangle = \langle y'|y\rangle$.

Tracing out the $\mathcal{H}_{\mathrm{anc},1}$ subsystem above gives

$$\mathrm{tr}_{\mathcal{H}_{\mathrm{anc},1}}\Big\{U_{\tilde{f}_s^*}(\rho \otimes \sigma)U_{\tilde{f}_s^*}^\dagger\Big\} = \sum_{t,t' \in S_{A^c}} a_{t,t'}\, \widetilde{O}^A_{t,t'} \otimes |t\rangle\langle t'| \sum_{y \in (A_0^{(r)})^{\otimes n'}} \sigma_{y,y} = \sum_{t,t' \in S_{A^c}} a_{t,t'}\, \widetilde{O}^A_{t,t'} \otimes |t\rangle\langle t'|\,. \quad (12)$$

Since we likewise have

$$\mathrm{tr}_{\mathcal{H}_{\mathrm{anc},1}}\Big\{U_{\tilde{f}_s^*}(\rho^0 \otimes \sigma^0)U_{\tilde{f}_s^*}^\dagger\Big\} = \sum_{t,t' \in S_{A^c}} a_{t,t'}\, O^A_{t,t'} \otimes |t\rangle\langle t'|\,, \quad (13)$$

we find that (12) and (13) agree upon taking the partial trace of each over $\mathcal{H}_A$. Thus we find that $\mathrm{tr}_{\mathcal{H}_{\mathrm{anc},1}}\Big\{U_{\tilde{f}_s^*}(\rho \otimes \sigma)U_{\tilde{f}_s^*}^\dagger\Big\}$ is (r,k)-deviated from $\mathrm{tr}_{\mathcal{H}_{\mathrm{anc},1}}\Big\{U_{\tilde{f}_s^*}(\rho^0 \otimes \sigma^0)U_{\tilde{f}_s^*}^\dagger\Big\}$, as claimed. □

4.A.5 Proof of super-polynomial separation between NISQ and BPP

We are now ready to complete the proof of the oracle separation between NISQ and BPP.

Proof of Theorem 2.2. We begin by showing hardness for BPP algorithms. Given a BPP algorithm for the robustified Simon's problem mapping n bits to $m^r n'$ bits, we show that we can produce a BPP algorithm for the original Simon's problem on n' bits with the same number of queries. Because $n' = 2^{\Theta(\log(n)^c)}$ for a constant $0 < c < 1$, this implies a super-polynomial query complexity lower bound of $\Omega(2^{2^{\Theta(\log(n)^c)}})$, as the classical query complexity of Simon's problem over n' bits is $\Omega(2^{n'/2})$ [25]. Indeed, by Eqs. (7), (8), whenever the BPP algorithm makes a query to the robustified Simon's oracle $\widetilde{f}_s$, we can simulate that with at most a single query to the standard Simon's oracle f_s, which immediately implies the desired simulation.

We now turn our attention to demonstrating a polynomial upper bound in NISQ. Let us decompose our total Hilbert space $\mathcal{H}$ as $\mathcal{H} \simeq \mathcal{H}_{\mathrm{main},1} \otimes \mathcal{H}_{\mathrm{main},2} \otimes \mathcal{H}_{\mathrm{anc},1} \otimes \mathcal{H}_{\mathrm{anc},2}$ where

$$\mathcal{H}_{\mathrm{main},1} \simeq (\mathbb{C}^2)^{\otimes(m^r n')},\ \mathcal{H}_{\mathrm{main},2} \simeq (\mathbb{C}^2)^{\otimes(n-m^r n')},\ \mathcal{H}_{\mathrm{anc},1} \simeq (\mathbb{C}^2)^{\otimes(m^r n')},\ \mathcal{H}_{\mathrm{anc},2} \simeq (\mathbb{C}^2)^{\otimes \mathcal{O}(\mathrm{poly}(n))}.$$

We begin with a state on $\mathcal{H}$ initialized in the all-zero state. By Lemma 4.A.4, we can prepare a state ρ on $\mathcal{H}_{\text{main},1}$, using the ancillas on $\mathcal{H}_{\text{anc},2}$, such that ρ is $(r, d/2)$-deviated from $\rho^0 = V^{\otimes n'} H^{\otimes n'} |0^{n'}\rangle\langle 0^{n'}| H^{\otimes n'} (V^{\otimes n'})^\dagger$. By Lemma 4.A.5, we can prepare a state σ on $\mathcal{H}_{\text{anc},1}$, using the ancillas on $\mathcal{H}_{\text{anc},2}$, such that σ is $(r, d/2)$-deviated from $\sigma^0 = V^{\otimes n'} |0^{n'}\rangle\langle 0^{n'}| (V^{\otimes n'})^\dagger$.

At this point in the algorithm, our qubits on $\mathcal{H}_{\text{main},2}$ are no longer in the all-zero state due to the local noise. We do not care what the state is and suppose that the state is given by $\rho^{(2)}$. We proceed by applying our oracle unitary $U_{\tilde{f}_s}$ to $(\rho \otimes \rho^{(2)}) \otimes \sigma$ on $\mathcal{H}_{\text{main},1} \otimes \mathcal{H}_{\text{main},2} \otimes \mathcal{H}_{\text{anc},1}$. Since the oracle unitary acts as the identity on $\mathcal{H}_{\text{main},2}$ by construction, we can equivalently just apply $U_{\tilde{f}_s^*}$ to $\rho \otimes \sigma$ on $\mathcal{H}_{\text{main},1} \otimes \mathcal{H}_{\text{anc},1}$. Doing so and subsequently neglecting the $\mathcal{H}_{\text{anc},1}$ register (corresponding to tracing out the qubits), we obtain

$$\rho' = \operatorname{tr}_{\mathcal{H}_{\text{anc},1}} \left\{ U_{\tilde{f}_s^*} (\rho \otimes \sigma) U_{\tilde{f}_s^*}^\dagger \right\} .$$

But by Lemma 4.A.7, this state is only $(r, d/2)$-deviated from

$$\rho^1 = \operatorname{tr}_{\mathcal{H}_{\text{anc},1}} \left\{ U_{\tilde{f}_s^*} (\rho^0 \otimes \sigma^0) U_{\tilde{f}_s^*}^\dagger \right\} .$$

If f_s is a 1-to-1 function, then

$$\rho^1 = V^{\otimes n'} \left(\frac{1}{2^{n'}} \sum_{z \in \{0,1\}^{n'}} |z\rangle\langle z| \right) (V^{\otimes n'})^\dagger ,$$

whereas if f_s is a 2-to-1 function we have

$$\rho^1 = V^{\otimes n'} \left(\frac{1}{2^{n'}} \sum_{z \in \{0,1\}^{n'}} \frac{1}{\sqrt{2}} \big(|z\rangle + |z \oplus s\rangle \big) \cdot \frac{1}{\sqrt{2}} \big(\langle z| + \langle z \oplus s| \big) \right) (V^{\otimes n'})^\dagger$$

where s is the hidden string. Applying Hadamards to the encoded qubits of ρ', measuring in the computational basis, and applying classical post-processing via recursive majority vote as per Lemma 4.A.6, we will obtain an n' bit string z_0 which with probability $1 - \mathcal{O}(1/n')$ is sampled from the distribution $\mathcal{D}$ defined as follows. If f_s is 1-to-1 function then $\mathcal{D}$ will be the uniform distribution over n' bit strings, whereas if f_s is a 2-to-1 function then $\mathcal{D}$ will be the uniform distribution over n' bit strings subject to the constraint $z_0 \cdot s = 0 \pmod 2$.

If we repeat the entire procedure n' times, then with probability $(1 - \mathcal{O}(1/n'))^{n'} = \Omega(1)$ we obtain n' such bit strings $z_0, z_1, ..., z_{n'-1}$. If this event, call it $\mathcal{E}$, happens, then by solving the n' linear equations $z_i \cdot s = 0 \pmod 2$ for $i = 0, 1, ..., n' - 1$, we can determine whether s is the all-zero string meaning f_s is 1-to-1, or some non-trivial string in which case f_s is 2-to-1. In general, if $\mathcal{E}$ does not happen and we have obtained some arbitrary string s, we can check that this situation is the case by querying the classical oracle at $f_s(0)$ and $f_s(s)$. So by repeating the entire procedure $\mathcal{O}(\log(1/\delta))$ times, with probability at least $1 - \delta$ the event $\mathcal{E}$ will happen at least once, and we will be able to determine if f_s is 1-to-1 or 2-to-1. □

Remark 4.A.1. *As alluded to at the beginning of this section, the proof above applies verbatim to the stronger noise model where at every layer, every qubit is independently corrupted with probability λ by an adversary. As a result, although our definition of* NISQ *pertains to local depolarizing noise, the oracle separation between* BPP *and* NISQ *holds even when the local noise could be adversarially chosen.*

4.B NISQ vs. BQP

In this section we show an oracle separation between NISQ and BQP via a simple "lifting" of Simon's problem. In fact, we will actually be able to separate NISQ and $\mathsf{BPP}^{\mathsf{QNC}^0}$ relative to this oracle.

We begin by describing the modification of Simon's problem we will consider. For $n \in \mathbb{N}$, given a function $f : \{0,1\}^n \to \{0,1\}^n$, we define the *lift* of f to be the function $\widetilde{f} : \{0,1\}^{2n} \to \{0,1\}^n$ given by

$$\widetilde{f}(x) \triangleq \begin{cases} f(x_1, \ldots, x_n) & \text{if } x_{n+1}, \ldots, x_{2n} = 0 \\ 0 & \text{otherwise} \end{cases}.$$

Given lifted function $\widetilde{f}$, we will abuse notation and let $O_{\widetilde{f}}$ denote both the classical oracle given by evaluating $\widetilde{f}$ as well as the quantum oracle

$$O_{\widetilde{f}} : |x\rangle\,|y\rangle \mapsto |x\rangle\,|y \oplus \widetilde{f}(x)\rangle\,.$$

It is not hard to see that in the absence of depolarizing noise, a minor modification of Simon's algorithm, which can be implemented in $\mathsf{BPP}^{\mathsf{QNC}^0}$, still works under this lifting. In contrast, for NISQ algorithms, we show the following:

Theorem 4.B.1. *Let $\lambda \in [0,1]$. Any NISQ_λ algorithm which, given oracle access to $O_{\widetilde{f}}$ for any lift of a function $f : \{0,1\}^n \to \{0,1\}^n$ which is either 2-to-1 or 1-to-1, can determine whether f is 2-to-1 or 1-to-1 with constant advantage must have query complexity at least $\exp(\Omega(\lambda n))$. Thus, relative to oracles O of this form, $\mathsf{NISQ}^O \subsetneq \mathsf{BQP}^O$.*

Our lifting operation is reminiscent of the shuffling Simon's problem introduced in [10] to give an oracle separation between $\mathsf{BPP}^{\mathsf{QNC}^0_d}$ and $\mathsf{BPP}^{\mathsf{QNC}^0_{2d+1}}$. As we show in Supplementary Note 8, the shuffling Simon's problem can also be used to separate NISQ from bounded-depth noiseless quantum computation. For instance, this implies the existence of an oracle relative to which $\mathsf{NISQ} \cup \mathsf{BPP}^{\mathsf{QNC}} \subsetneq \mathsf{BQP}$.

We now proceed to the proof of Theorem 4.B.1. We begin by recording the following basic fact about local depolarizing noise, whose proof we defer to Appendix 9.B.

Lemma 4.B.1. *Given $n' \in \mathbb{N}$, let Ω denote some subset of $\{0,1\}^{n'}$, and let Π denote the projection to the span of $\{|x\rangle\}_{x\in\Omega}$. Then for any $\lambda \in [0,1]$ and any n'-qubit state $|\psi\rangle$,*

$$\mathrm{tr}(\Pi D_\lambda^{\otimes n'}[|\psi\rangle\langle\psi|]) \le \sup_D \Pr_{a\sim D,\tilde{a}}[\tilde{a} \in \Omega], \tag{14}$$

where the supremum is over probability distributions over $\{0,1\}^{n'}$, and $\tilde{a}$ is the random string obtained by flipping each of the bits of a independently with probability $\lambda/2$.

Note the probability on the right-hand side of (14) is exponentially small when $\Omega \subset \{0,1\}^{2n}$ is the set of strings x for which $x_{n+1}, \ldots, x_{2n} = 0$. We will now use this to show that the distribution over measurement outcomes from running a noisy quantum circuit that has query access to either $O_{\widetilde{f}}$ or the identity oracle Id gives very little information about which oracle the circuit has access to.

Lemma 4.B.2. *Let A be any λ-noisy quantum circuit which makes N oracle queries. If $p_{\widetilde{f}}$ (respectively p_{id}) is the distribution over the random string s output by the circuit when the oracle is $O_{\widetilde{f}}$ (respectively the identity oracle* Id*), then $d_{TV}(p_{\widetilde{f}}, p_{\mathrm{id}}) \le N \exp(-\Omega(\lambda n))$.*

Proof. Let n' denote the number of qubits on which A operates. For convenience, we denote by $\widehat{O}_{\widetilde{f}}$ the channel given by pre-composing $O_{\widetilde{f}}$ with $D_\lambda^{\otimes 3n}$. We will show that for all n'-qubit pure states σ, $\|(\widehat{O}_{\widetilde{f}} \otimes D_\lambda^{\otimes n'-3n} - D_\lambda^{\otimes n'})[\sigma]\|_{\mathsf{tr}}$ is small so that we can apply Lemma 3.B.1.

When $\Omega \subset \{0,1\}^{2n}$ is given by all strings whose last n bits are 0, then for any $a \in \Omega$, if $\tilde{a}$ is obtained by flipping each of the bits of a independently with probability $\lambda/2$, then $\Pr[\tilde{a} \in \Omega] \le (1-\lambda/2)^n \le \exp(-\lambda n/2)$. So by Lemma 4.B.1, if $D_\lambda^{\otimes n'}[\sigma] = \sum_i \lambda_i |v_i\rangle\langle v_i|$, then $\sum_i \lambda_i \|\Pi' v_i\|^2 \le \exp(-\lambda n/2)$, where Π' is the projection to the span of $\{|x\rangle|y\rangle|w\rangle\}_{x\in\Omega, y\in\{0,1\}^n, w\in\{0,1\}^{n'-3n}}$. If we write every v_i as $\sum_{x\in\{0,1\}^{2n}, y\in\{0,1\}^n, w\in\{0,1\}^{n'-3n}} v_{i,x,y,w}|x\rangle|y\rangle|w\rangle$, then

$$\sum_i \lambda_i \sum_{x\in\Omega,y,w} v_{i,x,y,w}^2 \le \exp(-\lambda n/2).$$

and we see that $O_{\widetilde{f}} \otimes \mathrm{Id}$ maps $|v_i\rangle\langle v_i|$ to

$$\sum_{x,x'\in\Omega,y,y',w,w'} v_{i,x,y,w}v_{i,x',y',w'}|x, y\oplus f(g(x)), w\rangle\langle x', y'\oplus f(g(x')), w'|$$
$$+ \sum_{x\in\Omega,x'\notin\Omega,y,y',w,w'} v_{i,x,y,w}v_{i,x',y',w'}|x, y\oplus f(g(x)), w\rangle\langle x', y', w'|$$
$$+ \sum_{x\notin\Omega,x'\in\Omega,y,y',w,w'} v_{i,x,y,w}v_{i,x',y',w'}|x, y, w\rangle\langle x', y'\oplus f(g(x')), w'|$$
$$+ \sum_{x,x'\notin\Omega,y,y',w,w'} v_{i,x,y,w}v_{i,x',y',w'}|x, y, w\rangle|x', y', w'\rangle.$$

In particular,

$$\begin{aligned}
&\|(O_{\widetilde{f}} \otimes \mathrm{Id} - \mathrm{Id})[|v_i\rangle\langle v_i|]\|_{\mathsf{tr}} \\
&\quad\le \sqrt{2}\|(O_{\widetilde{f}} \otimes \mathrm{Id} - \mathrm{Id})[|v_i\rangle\langle v_i|]\|_F \\
&\quad\le 2\sqrt{2}\Big(\sum_{x\in\Omega \text{ or } x'\in\Omega,y,y',w,w'} v_{i,x,y,w}^2 v_{i,x',y',w'}^2\Big)^{1/2} \\
&\quad\le 2\sqrt{2}\Big(1-\Big(1-\sum_{x\in\Omega,y,w} v_{i,x,y,w}^2\Big)^2\Big)^{1/2} \le 4\sqrt{\sum_{x\in\Omega,y,w} v_{i,x,y,w}^2}.
\end{aligned}$$

By Jensen's inequality, we can bound $\|(\widehat{O}_{\widetilde{f}} \otimes D_\lambda^{\otimes n'-3n} - D_\lambda^{\otimes n'})[\sigma]\|_{\mathsf{tr}}$ by

$$4\sum_i \lambda_i \sqrt{\sum_{x\in\Omega,y,w} v_{i,x,y,w}^2} \le 4\sqrt{\sum_i \lambda_i \sum_{x\in\Omega,y,w} v_{i,x,y,w}^2} \le 4\exp(-\lambda n/4).$$

By taking the channels $\mathcal{E}_1$ and $\mathcal{E}_2$ in Lemma 3.B.1 to be $\widehat{O}_{\widetilde{f}} \otimes D_\lambda^{\otimes n'-3n}$ and $D_\lambda^{\otimes n'}$, we obtain the desired bound on $d_{\mathrm{TV}}(p_{\widetilde{f}}, p_{\mathrm{id}})$. □

We are ready to conclude the proof of Theorem 4.B.1. Roughly, because Lemma 4.B.2 tells us that running a noisy quantum circuit with oracle access gives negligible information about the underlying oracle, a NISQ algorithm with access to $O_{\widetilde{f}}$ is no more powerful than a classical algorithm with access to the corresponding classical oracle. The lower bound of Theorem 4.B.1 then follows from the classical lower bound for Simon's problem.

Proof of Theorem 4.B.1. Let $\mathcal{T}$ be the learning tree corresponding to a NISQ_λ algorithm that makes at most N classical or quantum oracle queries to $O_{\widetilde{f}}$, as in Definition 3.A.1. By Lemma 3.A.1 and Lemma 4.B.2, if we replace every noisy quantum circuit A in the tree with a noisy quantum circuit A' that makes queries to the identity oracle instead of to $O_{\widetilde{f}}$, then the new distribution over the leaves of $\mathcal{T}$ is at most $N^2\exp(-\Omega(\lambda n))$-far in total variation from the original distribution $p_{O_{\widetilde{f}}}$; for $N = \exp(o(\lambda n))$, this quantity is $o(1)$. For convenience, denote this new distribution by p'_f.

To apply Lemma 3.A.2, we wish to bound $d_{\mathrm{TV}}(\mathbb{E}_{f\text{ 1-to-1}}[p'_f], \mathbb{E}_{f\text{ 2-to-1}}[p'_f])$. But note that because the quantum circuits A' in the new learning tree are independent of the underlying function f, the learning tree is simply implementing a randomized classical query algorithm. We can thus think of p'_f as a mixture over distributions p'^r_f each corresponding to some fixing of the internal randomness r of the algorithm (here the coefficients of the mixture are independent of f). It thus suffices to bound $\sup_r d_{\mathrm{TV}}(\mathbb{E}_{f\text{ 1-to-1}}[p'^r_f], \mathbb{E}_{f\text{ 2-to-1}}[p'^r_f])$.

Henceforth fix any r. The rest of the argument follows the standard proof of the classical lower bound for Simons' algorithm. The algorithm queries the classical oracle at some deterministic sequence of inputs $x_1, \ldots, x_a$, which we may assume without loss of generality are distinct and lie in Ω. For any $y_1, \ldots, y_a$ which are all distinct, $(x_1, y_1), \ldots, (x_a, y_a)$ and r determine some leaf node ℓ of the tree. The probability of this leaf node under $\mathbb{E}_{f\text{ 1-to-1}}[p'^r_f]$ is $\frac{(2^n-a)!}{(2^n)!}$ and under $\mathbb{E}_{f\text{ 2-to-1}}[p'^r_f])$ is $\frac{M}{2^n-1}\frac{(2^n-a)!}{(2^n)!}$ where $M \triangleq 2^n - 1 - |\{x_i \oplus x_j \mid 1 \le i < j \le a\}|$. For any $y_1, \ldots, y_a$ for which there is a collision, the probability of the corresponding leaf node under $\mathbb{E}_{f\text{ 1-to-1}}[p'^r_f]$ is clearly 0. We conclude that the total variation between these two mixtures is upper bounded by the probability that there is a collision among $f(x_1), \ldots, f(x_a)$ for a random 2-to-1 function f. The latter is at most

$$\sum_{i=0}^{a-1} \frac{i}{2^n - 1 - \binom{i}{2}} \le \frac{a^2}{2^{n+1} - 2 - a^2},$$

so for $a \ll 2^{n/2}$, this quantity is $o(1)$. As $\min(\exp(\Omega(\lambda n)), 2^{n/2}) = \exp(\Omega(\lambda n))$, the theorem thus follows by Lemma 3.A.2. □

Remark 4.B.1. *The reader may observe that apart from the classical lower bound for Simon's problem, our proof of the lower bound in Theorem 4.B.1 makes very little use of the fact that f is either a 2-to-1 or 1-to-1 function. In fact, the above argument shows more generally that for any search problem over a family of Boolean functions, the query complexity of any* NISQ *algorithm for the lifted problem is essentially given by the classical query complexity for the original problem.*

Supplementary Note 5 – Unstructured Search

In this section, we show that there is no quadratic speedup for unstructured search in NISQ. Given $d \in \mathbb{N}$ and $i \in [d]$, we abuse notation and let O_i denote both the classical oracle $O_i : [d] \to \{0, 1\}$ given by $O_i(x) = \mathbb{1}[x = i]$ as well as the quantum oracle

$$O_i : |x\rangle |w\rangle \mapsto (-1)^{\mathbb{1}[x=i]} |x\rangle |w\rangle \quad \forall\, x \in [d].$$

We remark that we have opted to work with the phase version of the classical oracle for convenience, since the two standard formulations of the oracle can simulate one another and so our lower bound will be unaffected. We will also consider the identity oracle, which is classically given by $O_0(x) \triangleq x$ and quantumly given by $O_0 : |x\rangle |w\rangle \mapsto |x\rangle |w\rangle$.

Formally, we show the following:

Theorem 5..1. *Let $\lambda \in [0,1]$. Any* NISQ_λ *algorithm which, given oracle access to O_i for any $i \in [d]$, can determine i with probability $2/3$ must have query complexity at least $\widetilde{\Omega}(d\lambda)$.*

Our proof is composed of two parts. The first and main part is to establish a stronger result, namely a query complexity lower bound even for *noiseless* bounded-depth quantum computation. The second part is to verify, essentially via an argument of [22], that this implies a lower bound for NISQ.

5.A Proof preliminaries

We will still work with the tree formalism from Definition 3.A.1, but because our focus now is on noiseless bounded-depth quantum computation, the definition simplifies somewhat:

Definition 5.A.1 (Tree representation for bounded-depth algorithms)**.** *Given oracle O, a noiseless depth-T algorithm with access to O can be associated with a pair $(\mathcal{T}, \mathcal{A})$ as follows. The* learning tree *$\mathcal{T}$ is a rooted tree, where each node in the tree encodes the transcript of all classical query and noisy quantum circuit results the algorithm has seen so far. The tree satisfies the following properties:*

- *Each node u is associated with a value $p_O(u)$ corresponding to the probability that the transcript observed so far is given by the path from the root r to u. In this way, $\mathcal{T}$ naturally induces a distribution over its leaves; we abuse notation and denote this distribution by p_O. For the root r, $p_O(r) = 1$.*
- *At each non-leaf node u, we run a noiseless depth-T quantum circuit A with access to O. The children v of u are indexed by the possible $s \in \{0,1\}^{n'}$ that could be obtained as a result. We refer to the edge between u and v as (u, A, s). We denote by $|\phi_O(A)\rangle$ the output state of the circuit so that the probability of traversing (u, A, s) from node u to child v is given by $|\langle s|\phi_O(A)\rangle|^2$. We define*
$$p_O(v) = p_O(u) \cdot |\langle s|\phi_O(A)\rangle|^2 \,.$$
- *If the total number of queries to O made along any root-to-leaf path is at most N, we say that the* query complexity *of the algorithm is at most N.*

$\mathcal{A}$ is any classical algorithm that takes as input a transcript corresponding to any leaf node ℓ and attempts to determine the underlying oracle or predict some property thereof.

We note that one minor distinction between the tree formalism for bounded-depth computation versus the one for NISQ is that we do not consider classical queries to O. The reason is that because the quantum circuits A used at every node in Definition 5.A.1 are noiseless, we can simulate noiseless classical query access to O using quantum query access.

In the sequel, for $O = O_i$ for $i \in \{0, \ldots, d\}$, we will refer to p_O and ϕ_O in Definition 5.A.1 as p_i and ϕ_i.

We will make use of the following well-known bound:

Lemma 5.A.1 (Eq. (7) in [26])**.** *For any quantum circuit A for unstructured search that makes T oracle queries, if $|\phi_i(A)\rangle$ denotes the output state when the underlying oracle is O_i, then*

$$\sum_{i=1}^{d} \| |\phi_i(A)\rangle - |\phi_0(A)\rangle \|^2 \le 4T^2.$$

5.B Lower bound against bounded-depth computation

We now use these tools to prove the following query complexity lower bound. This will be the main component in our proof of Theorem 5..1.

Theorem 5.B.1. *There is an absolute constant $c > 0$ for which the following holds. Let $d, T \in \mathbb{N}$ with $T \leq d$. Then no noiseless quantum algorithm of depth T with query complexity at most cd/T can, given oracle access to O_i for any $i \in [d]$, output i with probability $2/3$.*

5.B.1 Likelihood ratio calculations

To prove Theorem 5.B.1, our goal is to bound $d_{\mathrm{TV}}(p_0, \mathbb{E}_{i\sim[d]} p_i)$. To that end, we will analyze the likelihood ratio between these two distributions. Given any path $\mathbf{z} = ((u_1, A_1, s_1), (u_2, A_2, s_2), \ldots, (u_T, A_T, s_T))$ in the tree, the likelihood ratio $L_i(\mathbf{z})$ between traversing that path when the underlying oracle is O_i versus when it is O_0 is given by

$$\begin{aligned} L_i(\mathbf{z}) &= \prod_{t=1}^{n} \frac{|\langle s_t|\phi_i(A_t)\rangle|^2}{|\langle s_t|\phi_0(A_t)\rangle|^2} \\ &\geq \prod_{t=1}^{n} \left[1 + \frac{\langle s_t|\phi_i(A_t)\rangle - \langle s_t|\phi_0(A_t)\rangle}{\langle s_t|\phi_0(A_t)\rangle} + \frac{\langle \phi_i(A_t)|s_t\rangle - \langle \phi_0(A_t)|s_t\rangle}{\langle \phi_0(A_t)|s_t\rangle}\right] \end{aligned}$$

For convenience, define

$$\begin{aligned} Y_i(u_t, A_t, s_t) &= \frac{\langle s_t|\phi_i(A_t)\rangle - \langle s_t|\phi_0(A_t)\rangle}{\langle s_t|\phi_0(A_t)\rangle} + \frac{\langle \phi_i(A_t)|s_t\rangle - \langle \phi_0(A_t)|s_t\rangle}{\langle \phi_0(A_t)|s_t\rangle} \\ &= 2\,\mathrm{Re}\left[\frac{\langle s_t|\phi_i(A_t)\rangle - \langle s_t|\phi_0(A_t)\rangle}{\langle s_t|\phi_0(A_t)\rangle}\right] \end{aligned}$$

so that we have

$$L_i(\mathbf{z}) \geq \prod_{t=1}^{n} \left(1 + Y_i(u_t, A_t, s_t)\right). \tag{15}$$

Our goal is to show that, with respect to the distribution over paths $\mathbf{z}$ when the underlying oracle is O_0, $\mathbb{E}_{i\sim[d]}[L_i(\mathbf{z})]$ is with high probability not too small. This readily implies the desired upper bound on $d_{\mathrm{TV}}(p_0, \mathbb{E}_{i\sim[d]} p_i)$, as the latter satisfies

$$d_{\mathrm{TV}}(p_0, \mathbb{E}_{i\sim[d]} p_i) = \mathop{\mathbb{E}}_{\mathbf{z}\sim p_0}[\max(0, 1 - \mathop{\mathbb{E}}_{i}[L_i(\mathbf{z})])] \leq \mathop{\mathbb{E}}_{\mathbf{z}\sim p_0, i}[\max(0, 1 - L_i(\mathbf{z}))].$$

The idea for showing this will be to argue that the successive partial products in (15) give rise to a *multiplicative sub-martingale*[2] with suitably bounded increments $1 + Y_i(u_t, A_t, s_t)$, so that we can apply off-the-shelf martingale concentration inequalities.

Key to bounding these increments are the following moment bounds on $Y_i(u, A, s)$, as a random variable in s.

Lemma 5.B.1. *For any edge (u, A, s) in the tree and any $i \in [d]$, we have that*

- $\mathbb{E}_s[Y_i(u, A, s)] = -\||\phi_i(A)\rangle - |\phi_0(A)\rangle\|_2^2$,
- $\mathbb{E}_s\left[Y_i(u, A, s)^2\right] \leq 4\||\phi_i(A)\rangle - |\phi_0(A)\rangle\|_2^2$,

[2] Equivalently, the partial sums $\sum_{t=1}^{m} \log(1 + Y_i(u_t, A_t, s_t))$ for $m = 1, \ldots, n$ give rise to a sub-martingale.

Here the expectations are with respect to the distribution over measurement outcomes when the underlying oracle is O_0.

Proof. Recalling that we observe outcome s under this distribution with probability $|\langle\phi_0(A)|s\rangle|^2$, we have

$$\begin{aligned}\mathop{\mathbb{E}}_s[Y_i(u,A,s)] &= \sum_s \langle\phi_0(A)|s\rangle\left(\langle s|\phi_i(A)\rangle - \langle s|\phi_0(A)\rangle\right) \\ &\quad + \sum_s \left(\langle\phi_i(A)|s\rangle - \langle\phi_0(A)|s\rangle\right)\langle s|\phi_0(A)\rangle \\ &= \langle\phi_0(A)|\,|\phi_i(A)\rangle - 1 + \langle\phi_i(A)|\,|\phi_0(A)\rangle - 1 \\ &= -\||\phi_i(A)\rangle - |\phi_0(A)\rangle\|^2,\end{aligned}$$

which establishes the first statement. For the second statement, we have

$$\begin{aligned}\mathop{\mathbb{E}}_s[Y_i(u,A,s)^2] &\le 4\mathop{\mathbb{E}}_s\left[\left|\frac{\langle s|\,|\phi_i(A)\rangle - \langle s|\,|\phi_0(A)\rangle}{\langle s|\,|\phi_0(A)\rangle}\right|^2\right] \\ &= 4\sum_s \left(\langle\phi_i(A)|s\rangle - \langle\phi_0(A)|s\rangle\right)\left(\langle s|\phi_i(A)\rangle - \langle s|\phi_0(A)\rangle\right) \\ &= 4\||\phi_i(A)\rangle - |\phi_0(A)\rangle\|_2^2.\end{aligned}$$

□

5.B.2 Good and balanced paths

In this section we define two conditions on paths $\mathbf{z}$ of the tree (Definitions 5.B.1 and 5.B.2) under which we can show that Eq. (15) is not too small with high probability over paths for which these two conditions hold. We then prove that a random path in $\mathcal{T}$ under p_0 will satisfy these conditions with high probability.

First, let $0 < \varepsilon < 1/2$ be a small enough constant that we will set later. Given a path in the learning tree given by $\mathbf{z} = ((u_1, A_1, s_1), \ldots, (u_n, A_n, s_n))$, if each A_t queries the oracle T_t times, define the potential

$$\tau(\mathbf{z}) \triangleq \sum_t T_t^2.$$

Note that if every algorithm in the tree queries the oracle T times, then $\tau(\mathbf{z}) = nT^2$; indeed, it may be helpful for the reader to focus on this case and think of $\tau(\mathbf{z})$ as nT^2 in the sequel. In general, Hölder's inequality implies the following basic fact:

Lemma 5.B.2. *If $(\mathcal{T}, \mathcal{A})$ specifies a noiseless quantum algorithm of depth T, then its query complexity is at least $\frac{1}{T}\max_{\mathbf{z}} \tau(\mathbf{z})$.*

We are now ready to define our two conditions on paths:

Definition 5.B.1. *We say that an edge (u, A, s) is i-*good *if*

$$Y_i(u, A, s) \ge -\varepsilon.$$

*We say that a path $\mathbf{z}$ is i-*good *if all of its constituent edges are i-good.*

Let $I_{\mathsf{good}}(\mathbf{z})$ (respectively $I_{\mathsf{bad}}(\mathbf{z})$) denote the set of indices $i \in [d]$ for which $\mathbf{z}$ is i-good (respectively not i-good).

Definition 5.B.2. *We say a path* $\mathbf{z}$ *is* i-balanced *if its constituent edges* $((u_1, A_1, s_1), \ldots, (u_n, A_n, s_n))$ *satisfy*

$$\sum_{t=1}^{n} \| |\phi_i(A_t)\rangle - |\phi_0(A_t)\rangle \|^2 \le \max_{\mathbf{z}'} \frac{40000}{d} \tau(\mathbf{z}').$$

Let $I_{\mathsf{bal}}(\mathbf{z})$ *(respectively* $I_{\mathsf{imbal}}(\mathbf{z})$*) denote the set of indices* $i \in [d]$ *for which* $\mathbf{z}$ *is* i*-balanced (respectively not* i*-balanced).*

Intuitively, goodness of a path ensures that as one goes from one partial product of (15) to the next, we never experience any significant multiplicative decreases. On the other hand, as we will see in the proof of Lemma 5.B.6 below, balancedness of a path ensures that the "variance" of these multiplicative changes is also not too large. These two conditions are important for applying off-the-shelf martingale concentration bounds like Freedman's inequality, which is governed by how large the changes can be in the worst case and how large they can be on average.

We now argue that most paths are i-good and i-balanced for most $i \in [d]$ (Lemmas 5.B.4 and 5.B.5 below). To do this, we will need the following consequence of Lemma 5.B.1.

Lemma 5.B.3. *For any edge* (u, A, s) *in the tree and any* $i \in [d]$*, we have that* $\Pr_s[(u, A, s) \text{ not } i\text{−good}] \le 12\| |\phi_i(A)\rangle - |\phi_0(A)\rangle \|_2^2/\varepsilon^2$*. Here the probability is with respect to the distribution over measurement outcomes when the underlying oracle is* O_0*.*

Proof. Suppose that $\| |\phi_i(A)\rangle - |\phi_0(A)\rangle \|^2 < \varepsilon/2$ (otherwise the claim vacuously holds). Then by Chebyshev's inequality,

$$\begin{aligned}
\Pr_s[(u, A, s) \text{ not } i\text{−good}] &= \Pr_s[Y_i(u, A, s) < -\varepsilon] \\
&\le \frac{\mathbb{V}_s[Y_i(u, A, s)]}{(\mathbb{E}_s[Y_i(u, A, s)] + \varepsilon)^2} \\
&\le \frac{3\| |\phi_i(A)\rangle - |\phi_0(A)\rangle \|_2^2}{(\varepsilon - \| |\phi_i(A)\rangle - |\phi_0(A)\rangle \|_2^2)^2} \\
&\le 12\| |\phi_i(A)\rangle - |\phi_0(A)\rangle \|_2^2/\varepsilon^2.
\end{aligned}$$

□

We now combine Lemma 5.A.1, Lemma 5.B.1, and Lemma 5.B.3 to conclude that for a root-to-leaf path $\mathbf{z}$ sampled according to p_0, the probability that $I_{\mathsf{good}}(\mathbf{z})$ or $I_{\mathsf{bal}}(\mathbf{z})$ is small is low.

Lemma 5.B.4. $\Pr_{\mathbf{z} \sim p_0}\left[|I_{\mathsf{good}}(\mathbf{z})| \le d - 4800\, \mathbb{E}_{\mathbf{z}' \sim p_0}[\tau(\mathbf{z})]/\varepsilon^2\right] \le 1/100$.

Proof. Note that

$$\begin{aligned}
\mathop{\mathbb{E}}_{\mathbf{z} \sim p_0}[|I_{\mathsf{bad}}(\mathbf{z})|] &= \sum_{i=1}^{d} \Pr_z[\mathbf{z} \text{ not } i\text{−good}] \\
&\le \sum_{i=1}^{d} \sum_{t=1}^{n} \Pr[(u_t, A_t, s_t) \text{ not } i\text{−good}] \\
&= \sum_{i=1}^{d} \sum_{t=1}^{n} \Pr_{s_t}[(u_t, A_t, s_t) \text{ not } i\text{−good}] \\
&\le \sum_{i=1}^{d} \sum_{t=1}^{n} \mathop{\mathbb{E}}_{\mathbf{z} \sim p_0}\left[12\| |\phi_i(A_t)\rangle - |\phi_0(A_t)\rangle \|^2/\varepsilon^2\right] \\
&\le \mathop{\mathbb{E}}_{z \sim p_0}[48\tau(\mathbf{z})/\varepsilon^2],
\end{aligned}$$

where the penultimate step follows by Lemma 5.B.3, and the last step follows by Lemma 5.A.1. The lemma follows by the fact that $|I_{\mathsf{bad}}(\mathbf{z})| + |I_{\mathsf{good}}(\mathbf{z})| = d$ and by Markov's inequality. □

Lemma 5.B.5. $\Pr_{\mathbf{z}\sim p_0}[|I_{\mathsf{bal}}(\mathbf{z})| < 99d/100] \le 1/100$.

Proof. Note that

$$\begin{aligned}
\mathop{\mathbb{E}}_{\mathbf{z}\sim p_0}[|I_{\mathsf{bal}}(\mathbf{z})|] &= \sum_{i=1}^{d}\Big(1 - \Pr_{\mathbf{z}}[\mathbf{z} \text{ not } i\text{-balanced}]\Big) \\
&\ge \sum_{i=1}^{d}\left(1 - \mathop{\mathbb{E}}_{\mathbf{z}\sim p_0}\left[\frac{\sum_{t=1}^{n}\||\phi_i(A_t)\rangle - |\phi_0(A_t)\rangle\|_2^2}{\max_{\mathbf{z}'} 40000\tau(\mathbf{z}')/d}\right]\right) \\
&\ge d - d \mathop{\mathbb{E}}_{\mathbf{z}\sim p_0}\left[\frac{\sum_{i=1}^{d}\sum_{t=1}^{n}\||\phi_i(A_t)\rangle - |\phi_0(A_t)\rangle\|^2}{\max_{\mathbf{z}'} 40000\tau(\mathbf{z}')}\right] \\
&\ge 9999d/10000.
\end{aligned}$$

where the second step follows by Markov's inequality and the last step follows by Lemma 5.A.1. The lemma follows by the fact that $|I_{\mathsf{bal}}(\mathbf{z})| + |I_{\mathsf{imbal}}(\mathbf{z})| = d$ and by Markov's inequality. □

5.B.3 Martingale concentration

The paths that are both i-balanced and i-good are the ones over which the log-likelihood $\log L_i(\mathbf{z})$ will concentrate as a random variable in $z \sim p_0$. As alluded to in the discussion above, being i-balanced ensures bounded variance, while being i-good ensures bounded differences. Together these yield the following Bernstein-type concentration which is the main technical ingredient in the proof of Theorem 5.B.1:

Lemma 5.B.6. *For any $i \in [d]$, consider the following sequence of random variables*

$$\Big\{X_t \triangleq \log\left(1 + Y_i(u_t, A_t, s_t)\right) \cdot \mathbb{1}[(u_t, A_t, s_t)\ i\text{−good}]\Big\}_{t=1}^{n},$$

where the randomness is with respect to p_0. For any $\eta, \nu > 0$, we have

$$\Pr_{\mathbf{z}}\left[\sum_{t=1}^{n} X_t \le -13\sum_{t=1}^{n}\||\phi_i(A_t)\rangle - |\phi_0(A_t)\rangle\|_2^2 - \eta \text{ and } \sum_{t=1}^{n}\||\phi_i(A_t)\rangle - |\phi_0(A_t)\rangle\|_2^2 \le \nu\right] \le \exp\left(-\frac{\eta^2}{16\nu + 4\varepsilon\eta/3}\right).$$

Proof. Note that for any (u, A, s),

$$\begin{aligned}
&\mathop{\mathbb{E}}_{s}[\log(1 + Y_i(u, A, s)) \cdot \mathbb{1}[(u, A, s)\ i\text{−good}]] \\
&\quad\ge \mathop{\mathbb{E}}_{s}\big[\big(Y_i(u, A, s) - Y_i(u, A, s)^2\big) \cdot \mathbb{1}[(u, A, s)\ i\text{−good}]\big] \\
&\quad\ge \mathop{\mathbb{E}}_{s}\big[Y_i(u, A, s) - Y_i(u, A, s)^2\big] - \mathop{\mathbb{E}}_{s}[Y_i(u, A, s) \cdot \mathbb{1}[(u, A, s) \text{ not } i\text{−good}]] \\
&\quad\ge -5\||\phi_i(A)\rangle - |\phi_0(A)\rangle\|_2^2 - \mathop{\mathbb{E}}_{s}[Y_i(u, A, s) \cdot \mathbb{1}[(u, A, s) \text{ not } i\text{−good}]] \\
&\quad\ge -5\||\phi_i(A)\rangle - |\phi_0(A)\rangle\|_2^2 - \mathop{\mathbb{E}}_{s}\big[Y_i(u, A, s)^2\big]^{1/2} \cdot \Pr_{s}[(u, A, s) \text{ not } i\text{−good}]^{1/2} \\
&\quad\ge -13\||\phi_i(A)\rangle - |\phi_0(A)\rangle\|_2^2.
\end{aligned} \tag{16}$$

where in the first step we used the fact that $\log(1+z) \geq z - z^2$ for $z \geq -1/2$, in the third step we used Lemma 5.B.1, in the fourth step we used Cauchy-Schwarz, and in the last step we used the last part of Lemma 5.B.1 and Lemma 5.B.3.

Additionally,

$$\begin{aligned}\mathop{\mathbb{E}}_{s}\left[\log(1+Y_i(u,A,s))^2 \cdot \mathbb{1}[(u,A,s)\ i\text{−good}]\right] &\leq \mathop{\mathbb{E}}_{s}\left[2Y_i(u,A,s)^2 \cdot \mathbb{1}[(u,A,s)\ i\text{−good}]\right] \\ &\leq \mathop{\mathbb{E}}_{s}\left[2Y_i(u,A,s)^2\right] \\ &= 8\||\phi_i(A)\rangle - |\phi_0(A)\rangle\|_2^2, \end{aligned} \tag{17}$$

where in the first step we used the fact that $\log(1+z)^2 \leq 2z^2$ for $z \geq -1/2$, and in the last step we used the second part of Lemma 5.B.1.

For every t, define the random variable

$$Z_t \triangleq \log\left(1+Y_i(u_t,A_t,s_t)\right) \cdot \mathbb{1}[(u_t,A_t,s_t)\ i\text{−good}] + 13\||\phi_i(A_t)\rangle - |\phi_0(A_t)\rangle\|_2^2,$$

where the randomness is with respect to p_0. By Eq. (16), $\{Z_t\}_t$ is a submartingale difference sequence satisfying $Z_t \geq \log(1-\varepsilon) \geq -2\varepsilon$ given $0 < \varepsilon < 1/2$, so the lemma follows by Freedman's inequality and Eq. (17). □

We will take η to be a small constant to be tuned later, and

$$\nu = (40000/d) \max_{\mathbf{z}} \tau(\mathbf{z}). \tag{18}$$

In light of Lemma 5.B.6, we introduce one more property of paths $\mathbf{z}$ which, together with goodness (Definition 5.B.1) and balancedness (Definition 5.B.2), ensures that $\mathbb{E}_{i\sim[d]}[L_i(\mathbf{z})]$ is not too small.

Definition 5.B.3. *We say that a root-to-leaf path $\mathbf{z}$ is i-concentrated if the sum as considered in Lemma 5.B.6 is not too negative, that is,*

$$\sum_{t=1}^{n} X_t > -13\sum_{t=1}^{n}\||\phi_i(A_t)\rangle - |\phi_0(A_t)\rangle\|^2 - \eta,$$

and/or the path is not i-balanced. Let $I_{\mathsf{conc}}(\mathbf{z})$ (respectively $I_{\mathsf{anticonc}}(\mathbf{z})$) denote the set of indices $i \in [d]$ for which $\mathbf{z}$ is i-concentrated (respectively not i-concentrated).

5.B.4 Completing the argument

We assume the query complexity of the algorithm is at most cd/T for a small constant $0 < c < 1$ to be tuned later. Note that by Lemma 5.B.2, this implies that

$$\max_{\mathbf{z}} \tau(\mathbf{z}) \leq cd. \tag{19}$$

Lemma 5.B.7. $\Pr_{\mathbf{z}\sim p_0}[|I_{\mathsf{conc}}(\mathbf{z})| < 99d/100] \leq 100\exp\left(-\frac{\eta^2}{640000c+4\varepsilon\eta/3}\right)$.

Proof. By Lemma 5.B.6, our choice of ν in Eq. (18), and (19), for any $i \in [d]$ we have

$$\Pr[\mathbf{z} \text{ is } i\text{-concentrated}] \geq 1 - \exp\left(-\frac{\eta^2}{640000c+4\varepsilon\eta/3}\right).$$

By linearity of expectation, $\mathbb{E}[|I_{\mathsf{anticonc}}(\mathbf{z})|] \leq d \cdot \exp\left(-\frac{\eta^2}{640000c+4\varepsilon\eta/3}\right)$, so the proof follows by the fact that $|I_{\mathsf{conc}}(\mathbf{z})| + |I_{\mathsf{anticonc}}(\mathbf{z})| = d$ and Markov's inequality. □

We are now ready to finish the argument.

Proof of Theorem 5.B.1. By taking

$$\eta = 1/10 \qquad c = 10^{-10} \qquad \varepsilon = 1/135,$$

we can combine Lemma 5.B.4, Lemma 5.B.5, and Lemma 5.B.7 to obtain

$$\Pr_{\mathbf{z}\sim p_0}\left[|I_{\mathsf{good}}(\mathbf{z})| < (99/100)(N/G)\right] \le 1/100,$$
$$\Pr_{\mathbf{z}\sim p_0}\left[|I_{\mathsf{bal}}(\mathbf{z})| < (99/100)(N/G)\right] \le 1/100,$$
$$\Pr_{\mathbf{z}\sim p_0}\left[|I_{\mathsf{conc}}(\mathbf{z})| < (99/100)(N/G)\right] \le 1/100.$$

By union bound, with probability at least 0.97 over $\mathbf{z} \sim p_0$, there are at least $0.97(N/G)$ indices i such that $\mathbf{z}$ is i-good, i-balanced, and i-concentrated. For an index i that satisfies all three conditions for a path $\mathbf{z}$, we have

$$\begin{aligned}
\log(L_i(\mathbf{z}) &\ge \sum_t \log(1 + Y_i(u_t, A_t, s_t)) = \sum_t X_t \\
&> -13\sum_{t=1}^{n} \| |\phi_i(A_t)\rangle - |\phi_0(A_t)\rangle \|^2 - \eta \\
&\ge -\frac{520000}{d}\max_{\mathbf{z}'} \tau(\mathbf{z}') - \eta \ge -1/10,
\end{aligned}$$

where in the second, third, and fourth steps we used that $\mathbf{z}$ is i-good, i-concentrated, and i-balanced respectively. Hence, $L_i(\mathbf{z}) \ge 9/10$ with probability at least 0.97^2 given a random $\mathbf{z} \sim p_0$ and random $i \in [d]$. Therefore, by Eq. 5.B.1 above, we can bound the total variation distance by

$$0.97^2(1 - 0.905) + (1 - 0.97^2) \le 1/6.$$

By Lemma 3.A.2, we conclude that provided the query complexity of the algorithm is at most cd/T, it cannot distinguish between the oracle O_0 and the oracle O_i for a random choice of i with constant advantage. □

5.C From bounded-depth to noisy computation

We now show how to extract from Theorem 5.B.1 a lower bound against NISQ. We begin with the following basic lemma, a proof of which we include in Appendix 9.A for completeness, that quantifies the amount of information that is lost from running many layers of noisy computation:

Lemma 5.C.1 (Lemma 8 from [22])**.** *Let A be a λ-noisy depth-T quantum circuit on n qubits with output state ρ. Then $\mathcal{I}(\rho) \triangleq n - S(\rho) \le (1-\lambda)^T \cdot n$, where $S(\cdot)$ denotes von Neumann entropy.*

We will also use the following standard operational characterization of $I(\rho)$:

Lemma 5.C.2 (See e.g. Lemma 2 from [22])**.** *Given any n-qubit state ρ and any POVM, the distributions p, q induced by respectively measuring ρ and $I/2^n$ with the POVM satisfy $KL(p\|q) \le \mathcal{I}(\rho)$.*

Proof of Theorem 5..1. Let $\mathcal{T}$ be the learning tree corresponding to a NISQ algorithm which has access to O_i for some $0 \le i \le d$ and has query complexity N, as in Definition 3.A.1. Let $\overline{T}$ be some choice of depth that we will tune later. We will convert $\mathcal{T}$ to a learning tree $\widehat{\mathcal{T}}$ corresponding to a bounded-depth noiseless NISQ algorithm, as in Definition 5.A.1.

Define $\widehat{\mathcal{T}}$ as follows. For every non-leaf node u, if the algorithm makes a single classical query at input j, then replace the edge $(u, x, O_i(x))$ to its child v by an edge (u, A, s) where A is a depth-1 quantum algorithm simulating the classical query. On the other hand, suppose that at u, the algorithm runs some λ-noisy quantum circuit A on $\mathrm{poly}(d)$ qubits. If A makes fewer than $\overline{T}$ oracle queries in total, then consider the noiseless quantum circuit A' which simulates A by applying depolarizing noise at each layer. If A makes more than $\overline{T}$ queries, then replace A with the quantum circuit A' that simply measures the maximally mixed state in the computational basis, rather than the output state $|\phi_i(A)\rangle$. By Lemma 5.C.2 and Pinsker's inequality, the total variation distance between the induced conditional distributions on children when $|\phi_i(A)\rangle$ gets measured versus when the maximally mixed state gets measured is at most $\sqrt{\frac{1}{2}\mathcal{I}(|\phi_i(A)\rangle)}$, and by Lemma 5.C.1 this is at most $(1-\lambda)^{\overline{T}/2} \cdot \mathcal{O}(\sqrt{\log d})$.

Let p_i (respectively $\widehat{p}_i$) denote the distribution over leaves when running the NISQ algorithm given by $\mathcal{T}$ (respectively the noiseless algorithm given by $\widehat{\mathcal{T}}$). As the number of oracle queries is at most N, the depth of both trees is at most N, so we conclude that the total variation distance between p_i and $\widehat{p}_i$ is at most $N(1-\lambda)^{\overline{T}/2} \cdot \mathcal{O}(\sqrt{\log d})$ by Lemma 3.A.1. We will take $\overline{T} = C\lambda^{-1}(\log\log d + \log N)$ for constant $C > 0$ so that this quantity is an arbitrarily small constant.

We conclude by Theorem 5.B.1 that if $N \le cd/\overline{T} = \Theta(d\lambda/(\log\log d + \log N))$, then the NISQ algorithm given by $\mathcal{T}$ cannot solve unstructured search with probability $2/3$. This concludes the proof of our $\widetilde{\Omega}(d\lambda)$ lower bound. □

Supplementary Note 6 – Bernstein-Vazirani Problem

In this section we show that a NISQ algorithm can solve the Bernstein-Vazirani problem [27] with $\mathcal{O}(\log n)$ queries, whereas it is known that any classical algorithm requires $\Theta(n)$ queries. As with the upper bound in Section 4.A, we will show that our algorithm is robust not just to local depolarizing noise, but also to arbitrary local noise that occurs with sufficiently small constant rate (see Remark 6.B.1).

We begin by recalling the Bernstein-Vazirani problem on n bits. There is an unknown function $f : \{0,1\}^n \to \{0,1\}$ of the form $f(x) = s \cdot x \pmod 2$, where $s \in \{0,1\}^n$ is often called the *hidden string*. The goal is to determine the hidden string. In the quantum context, the classical oracle is rendered into a unitary O_f which acts as

$$O_f : |x\rangle \otimes |y\rangle \mapsto |x\rangle \otimes |y \oplus f(x)\rangle$$

for $|x\rangle$ a state on n qubits and $|y\rangle$ a state on one qubit. In the noiseless quantum setting, the best quantum algorithm can find the hidden string s in $\Theta(1)$ queries [27].

We prove the following result on the NISQ complexity of the Bernstein-Vazirani problem:

Theorem 6..1. *Let $0 \le \lambda < 1/24$. Then there is a NISQ_λ algorithm which can solve the Bernstein-Vazirani problem with probability at least $1-\delta$ using $\mathcal{O}(\frac{1}{1-24\lambda}\log(n/\delta))$ queries.*

6.A Proof preliminaries

Let us establish some notation to be used in the proof.

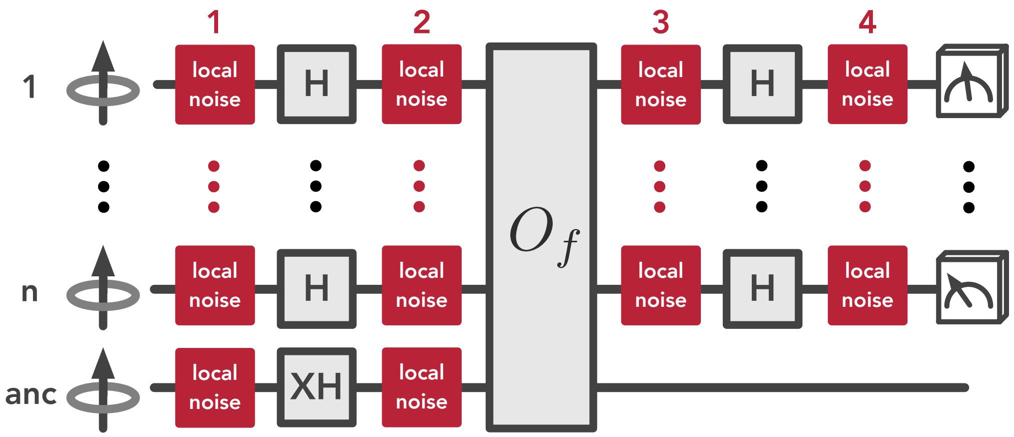

Supplementary Figure 2: Bernstein-Vazirani algorithm in the presence of arbitrary noise (each box labeled by "local noise" denotes that with probability λ, an arbitrary, adversarially chosen single-qubit operation is applied). We have labeled the layers of noise for ease of reference in the proof.

Definition 6.A.1 (Permutation operators)**.** *For $n \in \mathbb{N}$, let S_n be the permutation group on m objects. To each π in S_n we associate an operator acting on $(\mathbb{C}^2)^{\otimes n}$ defined by*

$$\pi\big(|\psi_1\rangle \otimes |\psi_2\rangle \otimes \cdots \otimes |\psi_n\rangle\big) = |\psi_{\pi^{-1}(1)}\rangle \otimes |\psi_{\pi^{-1}(2)}\rangle \otimes \cdots \otimes |\psi_{\pi^{-1}(n)}\rangle\,, \quad \forall\, |\psi_1\rangle, |\psi_2\rangle, ..., |\psi_n\rangle \in \mathbb{C}^2$$

which extends by multilinearity to all of $(\mathbb{C}^2)^{\otimes m}$.

We have a similar definition for permutations acting on bit strings.

Definition 6.A.2 (Permutations acting on bit strings)**.** *Again letting S_n be the permutation group on n objects, to each π in S_n we associate a function $\pi : \{0,1\}^n \to \{0,1\}^n$ defined by*

$$\pi(s_1 s_2 \cdots s_m) = s_{\pi^{-1}(1)} s_{\pi^{-1}(2)} \cdots s_{\pi^{-1}(m)}\,, \quad \forall s_1 s_2 \cdots s_m \in \{0,1\}^n\,.$$

Moreover, if $f : \{0,1\}^n \to \{0,1\}$ is the unknown function in the Bernstein-Vazirani problem, then we define $f_\pi := f \circ \pi$.

Bernstein-Vazirani algorithm. We conclude this subsection by reviewing how the original Bernstein-Vazirani algorithm [27] works, see Figure 2. One begins by preparing the initial state $|+\rangle^{\otimes n} \otimes |-\rangle$, and then acting on it with the oracle. In so doing, we obtain the state

$$\frac{1}{2^{n/2}} \sum_{x \in \{0,1\}^n} (-1)^{s \cdot x} |x\rangle \otimes |-\rangle\,.$$

Tracing out the $|-\rangle$ qubit, we can then apply the Hadamards $H^{\otimes n}$ to the first n qubits to obtain

$$\frac{1}{2^n} \sum_{x,y \in \{0,1\}^n} (-1)^{(s+y)\cdot x} |y\rangle = |s\rangle$$

which gives us the hidden string s.

6.B Proof of Theorem 6..1

With these notations at hand, we are ready to prove Theorem 6..1. Suppose we (noisily) initialize in the state $|+\rangle^{\otimes n} \otimes |-\rangle$. We call the last qubit the *ancilla qubit*. There are two cases depending on whether or not the ancilla qubit is corrupted prior to the oracle application in the algorithm. We handle these two cases separately in the following two lemmas.

Lemma 6.B.1. *If the ancilla qubit is not corrupted prior to application of the oracle in the Bernstein-Vazirani algorithm, then for every $i \in [n]$, with probability $(1-\lambda)^4$ the ith output bit is given by s_i and otherwise is given by a possibly incorrect bit.*

Proof. When we reach the step of the Bernstein-Vazirani algorithm where we apply the oracle, suppose that k qubits out of the first n have been corrupted in the first two layers of noise (see Figure 2). Further suppose that the qubits are located at $a_1, ..., a_k$, where $\{a_1, ..., a_k\} \subset \{1, ..., n\}$. Picking some permutation $\pi \in S_n$ such that $\pi(i) = a_i$ for $i = 1, ..., k$, we can write the state of the system as

$$\frac{1}{2^n} \sum_{\substack{z,z' \in \{0,1\}^k \\ x,x' \in \{0,1\}^{n-k}}} \beta_{z,z'}\, \pi(|z\rangle\langle z'| \otimes |x\rangle\langle x'|)\pi^\dagger \otimes |-\rangle\langle -|$$

for some coefficients $\{\beta_{z,z'}\}$ satisfying $\sum_z \beta_{z,z} = 1$. The rest of the protocol proceeds as follows. We apply O_f to get

$$\frac{1}{2^n} \sum_{\substack{z,z' \in \{0,1\}^k \\ x,x' \in \{0,1\}^{n-k}}} \beta_{z,z'}\, \pi(|z\rangle\langle z'| \otimes |x\rangle\langle x'|)\pi^\dagger \otimes |-\rangle\langle -|\, (-1)^{f_\pi(zx)+f_\pi(z'x')}\,.$$

Next we trace out the ancilla to find

$$\frac{1}{2^n} \sum_{\substack{z,z' \in \{0,1\}^k \\ x,x' \in \{0,1\}^{n-k}}} \beta_{z,z'} (-1)^{f_\pi(zx)+f_\pi(z'x')}\, \pi(|z\rangle\langle z'| \otimes |x\rangle\langle x'|)\pi^\dagger\,. \tag{20}$$

Following this we apply a third layer of local noise, apply $H^{\otimes n}$, and then apply a fourth layer of local noise again. Suppose that this procedure corrupts any number of the already corrupted qubits at positions $a_1, ..., a_k$, as well as ℓ qubits at positions different from $a_1, ..., a_k$. Suppose that the positions of these ℓ qubits are $a_{k+1}, ..., a_{k+\ell}$ where $\{a_1, ..., a_k, a_{k+1}, ..., a_{k+\ell}\} \subset \{1, ..., n\}$. Since the local noise and $H^{\otimes n}$ act qubit-wise, if we only want to track the uncorrupted qubits we can do as follows: at the outset we trace out the $k+\ell$ qubits which are to be corrupted, and then we apply $H^{\otimes(n-k-\ell)}$ to the residual qubits.

We implement this procedure presently. Defining $\sigma \in S_n$ by $\sigma(i) = a_i$ for $i = 1, ..., k+\ell$, we can rewrite (20) as

$$\frac{1}{2^n} \sum_{\substack{z,z' \in \{0,1\}^k \\ w,w' \in \{0,1\}^\ell \\ y,y' \in \{0,1\}^{n-k-\ell}}} \beta_{z,z',w,w'} (-1)^{f_\sigma(zwy)+f_\sigma(z'w'y')}\, \sigma(|z\rangle\langle z'| \otimes |w\rangle\langle w'| \otimes |y\rangle\langle y'|)\sigma^\dagger$$

for some coefficients $\{\beta_{z,z',w,w'}\}$ satisfying $\sum_{z,w} \beta_{z,z,w,w} = 1$. Letting $\{b_1, ..., b_{n-k-\ell}\} = \{1, ..., n\} \setminus \{a_1, ..., a_{k+\ell}\}$ be the uncorrupted registers, where we choose $b_1 < b_2 < \cdots < b_{n-k-\ell}$, tracing out everything but the $b_1, ..., b_{n-k-\ell}$ registers we find the residual pure state

$$\frac{1}{\sqrt{2^{n-k-\ell}}} \sum_{y \in \{0,1\}^{n-k-\ell}} (-1)^{y \cdot [s]_{b_1,...,b_{n-k-\ell}}}\, |y\rangle$$

where $[s]_{b_1,...,b_{n-k-\ell}}$ denotes the $b_1, ..., b_{n-k-\ell}$ bits of the hidden string s. Applying $H^{\otimes(n-k-\ell)}$ we find

$$\frac{1}{2^{n-k-\ell}} \sum_{y,y' \in \{0,1\}^{n-k-\ell}} (-1)^{y \cdot ([s]_{b_1,...,b_{n-k-\ell}} + y')} |y'\rangle \,.$$

Finally, measuring in the computational basis, the probability of measuring $|[s]_{b_1,...,b_{n-k-\ell}}\rangle$ is equal to one.

All in all, we have seen that if we perform the usual Bernstein-Vazirani algorithm, we obtain the hidden bit string s but with a fraction of its bits corrupted, corresponding precisely to the qubits that were corrupted by one of the four layers of local noise. □

We can now conclude the proof of Theorem 6..1.

Proof of Theorem 6..1. By Lemma 6.B.1, we see that for each bit i of the n output bits, the probability of being $|s_i\rangle$ is at least $(1-\lambda)^6$, which happens if the ancilla qubit is never corrupted in either of the two layers of noise prior to the application of the oracle (with probability $(1-\lambda)^2$), and if additionally the former of the two possible events in Lemma 6.B.1 happens (with probability $(1-\lambda)^4$). Let $f(\lambda) \triangleq (1-\lambda)^6$ and note that for $\lambda \leq 1/10$, $f(\lambda) > 1/2$.

Let X_i be a random variable which equals zero if s_i is obtained correctly with our procedure, and equal to one otherwise. Letting Y_i be the average of M i.i.d. copies of X_i, then the Chernoff-Hoeffding bound tells us that

$$\text{Prob}\left(Y_i^{(j)} \geq \frac{1}{2}\right) \leq \exp\left(-2M\left(\frac{1}{2} - f(\lambda)\right)^2\right) \,.$$

This is an upper bound on the probability that if we repeat the Bernstein-Vazirani algorithm M times and employ the majority votes strategy on the ith site to determine s_i, then we will fail. The probability that we fail for at least one of the n sites is upper bounded by

$$\text{Prob}\left(\max_i Y_i \geq \frac{1}{2}\right) \leq \sum_{i=1}^{n} \exp\left(-2M\left(\frac{1}{2} - f(\lambda)\right)^2\right) \leq n \exp\left(-2M\left(\frac{1}{2} - f(\lambda)\right)^2\right) \,.$$

So if we want the right-hand side to be at most δ, then we can pick some M such that

$$M = O\left(\frac{1}{(1 - 2\,f(\lambda))^2} \log(n/\delta)\right) \,. \tag{21}$$

Since $(1-2f(\lambda))^2$ is an alternating series in λ, we can lower bound it by its first two terms as

$$(1 - 2f(\lambda))^2 \geq 1 - 24\lambda \,.$$

Then (21) can be written in a slightly simplified form as

$$M = \left(\frac{1}{1 - 24\lambda} \log(n/\delta)\right)$$

for $\lambda < 1/24$, as claimed.[3] □

[3]We have not attempted to optimize this threshold for λ for general local noise channels or particular choices of local noise channels. However, we note that with more careful bookkeeping one can show, e.g. when the local noise is depolarizing noise, that for any λ bounded away from 1 the above algorithm can solve the Bernstein-Vazirani problem with $\mathcal{O}(\log(n/\delta))$ queries.

Remark 6.B.1. *As with the proof of Theorem 2.2, our proof considers a stronger noise model where at every layer, every qubit is independently corrupted with probability λ by a (potentially adversarially chosen) single-qubit channel. While our definition of* NISQ *focuses on local depolarizing noise, the quantum advantage in solving the Bernstein-Vazirani problem holds even when the local noise could be adversarially chosen.*

Supplementary Note 7 – Shadow Tomography

In this section, we show that relative to a natural *quantum oracle*, there is also an exponential separation even between NISQ and $\mathsf{BPP}^{\mathsf{QNC}^0}$. The task we consider that witnesses this separation has been studied previously in the context of separations between algorithms with and without quantum memory [28, 23, 29] and is based on *shadow tomography*, i.e. predicting properties of an unknown state to which one has access via a state oracle.

The shadow tomography problem, originally posed in [30], asks the following: given observables $O_1, \ldots, O_m$ and copies of an unknown n-qubit quantum state ρ, estimate each $\operatorname{tr}(O_i\rho)$ to small additive error. With arbitrary noiseless quantum computation, it is known that $\operatorname{poly}(\log m, n, 1/\varepsilon)$ copies suffice [30, 31, 32]; notably, the dependence on d is exponentially smaller than what is needed for full state tomography. These protocols need to make measurements that are entangled across multiple copies of ρ, and a separate line of work has provided alternative protocols that only measure a single or a small number of copies at a time [33, 28], as well as nearly matching lower bounds [28, 23] showing that such protocols cannot achieve the same statistical rates as those that use entangled measurements.

Here, we focus on the well-studied special case of *Pauli shadow tomography* where the observables consist of n-qubit Pauli operators. Informally, for an unknown state ρ, one would like to predict $|\operatorname{tr}(Q\rho)|$ for all Pauli operators $Q \in \{I, X, Y, Z\}^{\otimes n}$ given copies of ρ. We will show that NISQ_λ algorithms with access to such an oracle require $1/(1-\lambda)^{\Omega(n)}$ copies of ρ to estimate all of these observables to within constant error. On the other hand, existing upper bounds [28] imply that $\mathsf{BPP}^{\mathsf{QNC}^0}$ algorithms only require $\mathcal{O}(n)$ copies of ρ.

The Pauli shadow tomography task was the basis of an exponential separation [23] between learning algorithms with and without quantum memory which was demonstrated empirically on Google's Sycamore processor [29]. The distinction between these results and the lower bound we prove in this section is that the former considers noiseless quantum computation, whereas our bound pertains specifically to the NISQ setting. A matching upper bound in a more general noisy setting was shown in [34].

7.A A quantum oracle preparing an unknown state

Before stating this separation formally in Theorem 7.B.1 below, we first formalize how to extend the oracle model from Section 2.A to quantum oracles. We consider noisy quantum circuits with two registers: an n-qubit state register for loading in new copies of ρ, and a separate workspace register on at most $\operatorname{poly}(n)$ qubits.

Definition 7.A.1. *Let ρ be an n-qubit state. We consider an oracle O_ρ given as a CPTP map that traces out n qubits in the* state *register and prepares the state ρ in the* state *register:*

$$O_\rho(\sigma) \triangleq \rho \otimes \operatorname{tr}_{\mathsf{state}}(\sigma),$$

for any integer $n' \geq n$ and any n'-qubit state σ.

For the purposes of showing a lower bound in this oracle model, we will assume that in between oracle queries, the algorithm can perform arbitrary noiseless quantum computation, and at the end it can perform a noiseless measurement in the computational basis. The only noise that gets applied is local depolarizing noise after any call to O_ρ. Note that this is a stronger model of computation than a λ-noisy quantum algorithm or a NISQ_λ algorithm, which merely makes the lower bound we show even stronger. Furthermore, as there is no notion of a classical oracle in this setting, it is not necessary to work with the tree formalism of Definition 3.A.1.

7.B Exponential lower bound

We are now ready to state the main oracle separation of this section:

Theorem 7.B.1. *Let $\rho = \frac{1}{2^n}(I + s \cdot P)$ for some $s \in \{0,1\}$ and n-qubit Pauli operator P. Given access to the oracle O_ρ in Definition 7.A.1, no NISQ_λ algorithm can determine either s or P with constant advantage unless it makes $\Omega((1-\lambda)^{-n})$ oracle queries.*

On the other hand, there is an algorithm in $\mathsf{BPP}^{\mathsf{QNC}^0}$ that, given $\mathcal{O}(n)$ oracle queries, can determine both s and P with high probability. In fact, even if ρ is an arbitrary state, there is an algorithm in $\mathsf{BPP}^{\mathsf{QNC}^0}$ that, given $\mathcal{O}(n)$ oracle queries, can estimate $|\operatorname{tr}(P\rho)|$ for all n-qubit Pauli operators P to within small constant error with high probability.

We remark that [34] gives an upper bound of $(1-\lambda)^{-\Theta(n)}$ for NISQ_λ algorithms, so our exponential lower bound is qualitatively best possible. For the proof of Theorem 7.B.1 we consider the output state of any noisy quantum circuit and argue that for any Pauli operator P, the distance between the output state when the unknown state ρ is given by $\frac{1}{2^n}(I+P)$ (likewise when it is given by $\frac{1}{2^n}(I-P)$) versus when it is given by $\frac{I}{2^n}$ is exponentially small unless if the circuit makes exponentially many oracle queries:

Lemma 7.B.1. *For any $\lambda > 0$. Let $P \in \{I, X, Y, Z\}^{\otimes n}$. Any NISQ_λ algorithm that has oracle access to the state oracle O_ρ for either $\rho = \frac{1}{2^n}(I + P)$ or $\rho = \frac{I}{2^n}$ and can distinguish which oracle it has access to with at least $2/3$ probability must make $\Omega((1-\lambda)^{-|P|})$ queries, where $|P|$ denotes the number of non-identity components in P.*

Proof. Suppose the circuit operates on n' qubits. For convenience, for $s \in \{0,1\}$ denote $O_{\frac{1}{2^n}(I+s\cdot P)}$ by O_s. We would like to show that for all n'-qubit states σ, $\|D_\lambda^{\otimes n'}[(O_1 - O_0)[\sigma]]\|_{\mathsf{tr}}$ is small so that we can apply Lemma 3.B.1. But note that for any $Q \in \{X, Y, Z\}$, $D_\lambda[Q] = (1-\lambda)Q$, whereas $D_\lambda[I] = I$. We conclude that

$$\|D_\lambda^{\otimes n'}[(O_1 - O_0)[\sigma]]\|_{\mathsf{tr}} = \left\| D_\lambda^{\otimes n'}\left[\frac{P}{2^n} \otimes \operatorname{tr}_{\mathsf{state}}(\sigma)\right]\right\|_{\mathsf{tr}} \leq \left\| D_\lambda^{\otimes n}\left[\frac{P}{2^n}\right]\right\|_{\mathsf{tr}} = (1-\lambda)^{|P|}.$$

By taking the channels $\mathcal{E}$ in Lemma 3.B.1 to be $D_\lambda^{\otimes n'} \circ O_1$ and $D_\lambda^{\otimes n'} \circ O_0$, we conclude that no algorithm given by alternately querying the oracle followed by depolarizing noise, and running arbitrary noiseless quantum computation, and finally measuring in the computational basis can distinguish whether the underlying oracle is O_0 or O_1 with at least $2/3$ probability unless it makes $\Omega((1-\lambda)^{-|P|})$ queries.

As this model is a stronger model of computation than NISQ_λ (note that there is no notion of a classical oracle in this setting), this implies the claimed lower bound for NISQ_λ. □

Theorem 7.B.1 follows immediately from Lemma 7.B.1:

Proof of Theorem 7.B.1. The second part of the theorem was shown in [28, Theorem 2]. The NISQ_λ part of the theorem follows by applying Lemma 7.B.1 with P ranging over all n-qubit Pauli operators which act nontrivially on all qubits. The Lemma implies that regardless of which P is the one defining the oracle, the algorithm is unable to distinguish whether it has access to O_ρ or $O_{I/2^n}$ without making $\Omega((1-\lambda)^{-n})$ queries. □

Supplementary Note 8 – Separating NISQ and $\mathsf{BPP}^{\mathsf{QNC}}$ from BQP

While the relatively simple oracle from Section 4.B allowed us to separate NISQ and BQP, it is insufficient even to separate $\mathsf{BPP}^{\mathsf{QNC}^0}$ from BQP, as Simon's algorithm can also be implemented in the former. Here we show how to simultaneously separate NISQ and $\mathsf{BPP}^{\mathsf{QNC}}$ from BQP, at the cost of relying on a more involved oracle construction, namely the shuffling Simon's problem from [10].

We first recall the setup of the shuffling Simon's problem.

Definition 8..1 (Shuffling). *Given a function $f : \{0,1\}^n \to \{0,1\}^n$, consider any sequence of functions $f_0, \ldots, f_d : \{0,1\}^{(d+2)n} \to \{0,1\}^{(d+2)n}$ where $f_0, \ldots, f_{d-1}$ are 1-to-1 functions, and where f_d is chosen as follows. Define $S_d \subset \{0,1\}^{(d+2)n}$ to be the set of all $f_{d-1} \circ \cdots \circ f_0(x)$ for x ranging over the lexicographically first 2^n bitstrings in $\{0,1\}^{(d+2)n}$. Then define*

$$f_d(x) = \begin{cases} f((f_{d-1} \circ \cdots \circ f_0)^{-1}(x)) & \text{if } x \in S_d \\ \mathbf{0} & \text{otherwise} \end{cases},$$

where we identify the lexicographically first 2^n bitstrings in $\{0,1\}^{(d+2)n}$ with $\{0,1\}^n$ in the natural way.[4] *Let $\mathbf{SHUF}(f,d)$ denote the set of all sequences $(f_0, \ldots, f_d)$ that can be obtained in this way, and let $\mathcal{D}(f,d)$ denote the distribution over $\mathbf{SHUF}(f,d)$ induced by sampling $f_0, \ldots, f_{d-1}$ uniformly at random from the set of 1-to-1 functions.*

We now describe the quantum oracle associated to $\mathbf{SHUF}(f,d)$.

Definition 8..2 (Quantum shuffling oracle). *Given $(f_0, \ldots, f_d) \in \mathbf{SHUF}(f,d)$, we define the associated quantum channel $\mathcal{F}$ as follows. Given input state*

$$|\phi\rangle = \bigotimes_{i=0}^{d} |i, x_i\rangle\, |y_i\rangle\,,$$

where $x_0, \ldots, x_d \in \{0,1\}^{(d+2)n}$, we define

$$\mathcal{F}\,|\phi\rangle \triangleq \bigotimes_{i=0}^{d} |i, x_i\rangle\, |y_i \oplus f_i(x_i)\rangle\,.$$

The shuffling oracle *$O_{f,d}$ then sends $|\phi\rangle$ to the mixed state*

$$O_{f,d}(|\phi\rangle\langle\phi|) \triangleq \mathop{\mathbb{E}}_{\mathcal{F}\sim\mathcal{D}(f,d)}[\mathcal{F}|\phi\rangle\langle\phi|\mathcal{F}].$$

We are now ready to describe the shuffling Simon's problem.

[4] In [10], they define $f_d(x) = \perp$ for all $x \notin S_d$ for a special output symbol $\perp$. The shuffling Simon's problem under our definition is strictly more difficult, so the lower bound they prove immediately translates to a lower bound in our setting.

Definition 8..3 (d-Shuffling Simon's Problem: d-SSP)**.** *Let $f : \{0,1\}^n \to \{0,1\}^n$ be a random 2-to-1 function with probability 1/2, and a random 1-to-1 function otherwise. Given quantum access to the oracle $O_{f,d}$ as well as classical oracle access to f, the problem is to decide which of these two cases we are in.*

The lower bound against $\mathsf{BPP}^{\mathsf{QNC}}$ is immediate from [10]:

Theorem 8..1 (Corollary 1.3 from [10])**.** *For $d = \log^{\omega(1)}(n)$, d-$\mathsf{SSP} \in \mathsf{BQP}^O$ but d-$\mathsf{SSP} \notin (\mathsf{BPP}^{\mathsf{QNC}})^O$, where O denotes the shuffling oracle $O_{f,d}$ of the underlying function f.*

The main result of this section is to show that d-SSP also yields an oracle separation between BQP and NISQ_λ.

Theorem 8..2. *Any NISQ_λ algorithm that solves d-SSP with constant advantage must make $\exp(\Omega(n \cdot \min(1/2, \lambda d)))$ oracle queries.*

The lower bound against NISQ follows along similar lines to the argument in Section 4.B. The primary difference is that the subset Ω from Section 4.B was chosen to consist of all strings where the last n bits are 0, whereas the subset S_d of the domain on which f_d acts nontrivially in Definition 8..1 is a random subset. We will argue that with high probability over the randomness of this subset, depolarizing noise will kill off most of a states' component within the subspace corresponding to this subset.
We begin with a simple lemma showing that random small subsets of the hypercube are well-separated. This lemma is merely a re-interpretation of the Gilbert-Varshamov bound.

Lemma 8..1. *Let Ω be a uniformly random subset of $\{0,1\}^M$ of size S, where $|S| \le 2^{M-1}$. Then the probability that there exist distinct $x, y \in \Omega$ that are $\frac{M}{2}(1 - \sqrt{2\log_2(S^2/\delta)/M})$-close in Hamming distance is at most δ.*

Proof. Given $x \in \{0,1\}^M$ and $0 \le r \le M$, let $B(x,r)$ denote the Hamming ball around x of radius r. Note that

$$|B(x,r)| = \sum_{i=0}^{r} \binom{M}{i} \le 2^{M \cdot H(r/M)},$$

where $H(\cdot)$ denotes the binary entropy function. So given a subset $\Omega' \subset \{0,1\}^M$ of size S', the probability that a random point from $\{0,1\}^M \backslash \Omega'$ is at least r-far in Hamming distance from every point of Ω' is

$$1 - \frac{2^{M \cdot H(r/M)} \cdot S'}{2^M - S'}.$$

As we can sample a random subset Ω of $\{0,1\}^M$ of size S by randomly sampling S points from $\{0,1\}^M$ without replacement, we conclude that the probability that these points are all r-far in Hamming distance is

$$\begin{aligned}\prod_{t=0}^{S-1}\left(1 - \frac{2^{M \cdot H(r/M)} \cdot t}{2^M - t}\right) &\ge 1 - 2^{M \cdot H(r/M)} \sum_{t=0}^{S-1} \frac{t}{2^M - t} \ge 1 - S^2 \cdot 2^{-M(1-H(r/M))} \\ &\ge 1 - S^2 \cdot 2^{-M(1-2\sqrt{r/M - r^2/M^2})}.\end{aligned}$$

By taking $r = \frac{M}{2}(1 - \sqrt{2\log_2(S^2/\delta)/M})$, we find that the probability that all points in Ω are at least r far in Hamming distance is at least $1 - \delta$ as claimed. ☐

We would like to apply Lemma 4.B.1 to Ω consisting of strings which are separated in Hamming distance. To that end, we prove the following expansion result for such subsets of the hypercube.

Lemma 8..2. *Let $3/10 \le q < 1/2$. Let $\Omega \subset \{0,1\}^n$ be a subset such that all strings are at least qn apart in Hamming distance. Then for any distribution D over $\{0,1\}^n$ and any $0 < \lambda \le 1$, if $\tilde{a}$ is obtained by sampling $a \sim D$ and independently flipping each bit of a with probability $\lambda/2$, then $\Pr[\tilde{a} \in \Omega] \le \exp(-\Omega(\lambda n))$.*

Proof. Consider any fixed string $a \in \{0,1\}^n$. If the Hamming distance from a to any element of Ω is at least $qn/2$, then conditioned on a, the probability that $\tilde{a} \in \Omega$ is at most $(\lambda/2)^{qn/2}(1-\lambda/2)^{n(1-q/2)} \cdot |\Omega| \le 2^{-qn/2} \cdot |\Omega|$. On the other hand, if the Hamming distance from a to some element $x \in \Omega$ is some $d \le qn/2$, then by triangle inequality its Hamming distance to any other element of Ω is at least $qn - d \ge qn/2$. Conditioned on such an a, the probability that $\tilde{a} \in \Omega$ can be bounded by

$$\Pr[\tilde{a} = x \mid a] + (\lambda/2)^{qn/2}(1-\lambda/2)^{n(1-q/2)} \cdot (|\Omega - 1|) \le (1-\lambda/2)^n + 2^{-qn/2} \cdot |\Omega|.$$

Putting everything together, we conclude that

$$\Pr[\tilde{a} \in \Omega] \le \exp\left(n \cdot \left[2(1/2-q)^2 - (qn/2)\ln 2\right]\right) + \exp(-\lambda n/2).$$

Note that if $q \ge 0.28$, this expression is at most $\exp(-Cn)$ for an absolute constant $C > 0$. □

Combining Lemma 4.B.1 and Lemma 8..2 immediately yields the following estimate:

Corollary 8..1. *Let $3/10 \le q < 1/2$. Given $\Omega \subset \{0,1\}^n$ such that all strings in Ω are at least qn apart in Hamming distance, let Π denote the projection to the span of $\{|x\rangle\}_{x\in\Omega}$. Then for any $0 < \lambda \le 1$ and any n-qubit pure state $|\psi\rangle$,*

$$\mathrm{tr}\left(\Pi D_\lambda[|\psi\rangle\langle\psi|]\right) \le \exp(-\Omega(\lambda n)).$$

We now show the analogue of Lemma 4.B.2 for d-SSP:

Lemma 8..3. *Let A be any λ-noisy quantum circuit which makes N oracle queries to $O_{f,d}$ for some $f : \{0,1\}^n \to \{0,1\}^n$. If $p_{f,d}$ is the distribution over the random string s output by the circuit when the oracle is $O_{f,d}$, then $d_{TV}(p_{f,d}, p_{f',d}) \le \exp(-\Omega(\lambda dn))$ for any $f, f' : \{0,1\}^n \to \{0,1\}^n$.*

Proof. For convenience, define $n' \triangleq (d+2)n$, and suppose that A operates on n'' qubits. Let $f : \{0,1\}^n \to \{0,1\}^n$ be an arbitrary Boolean function. We would like to show that for all n''-qubit pure states σ, $\|(O_{f,d} \otimes \mathrm{Id} - \mathrm{Id})[D_\lambda^{\otimes n''}[\sigma]]\|_{\mathsf{tr}}$ is small so that we can apply Lemma 3.B.1.

Given $\mathcal{F} \in \mathbf{SHUF}(f,d)$, let $\Pi_{\mathcal{F}}$ denote the projection to the span of $\{|x\rangle\}_{x\in S_d}$ for the set S_d given by $\mathcal{F}$; we will occasionally denote S_d by $S_d(\mathcal{F})$ to emphasize the dependence on $\mathcal{F}$. For $\mathcal{F} \sim \mathcal{D}(f,d)$, observe that S_d is a uniformly random subset of $\{0,1\}^{n'}$ of size 2^n, so by Lemma 8..1, we have with probability at least $1 - 2^{-cdn}$ that all strings in S_d are at least Hamming distance $\Delta \triangleq \frac{n'}{2}\big(1 - \frac{(4+2cd)n}{n'}\big)^2$ apart. By taking c a sufficiently small absolute constant, we note that $\Delta \ge 3n'/10$ provided d is larger than some absolute constant.

Let $E \subseteq \mathbf{SHUF}(f,d)$ denote the set of $\mathcal{F}$ for which this is the case, so that $|E|/|\mathbf{SHUF}(f,d)| \ge 1 - 2^{-c}$. By Lemma 8..1 (applied to n'-qubit states instead of n-qubit states), $\mathrm{tr}(\Pi_{\mathcal{F}} D_\lambda^{\otimes n'}[\rho]) \le \exp(-\lambda n'/2) + \exp(-Cn')$ for an absolute constant $C > 0$ for all $\mathcal{F} \in E$ and n'-qubit pure states ρ.

If $D_\lambda^{\otimes n''}[\sigma] = \sum_j \lambda_j |\phi_j\rangle |\phi_j\rangle$ for

$$|\phi_j\rangle = \sum_{\mathbf{x},\mathbf{y}} v_{j,\mathbf{x},\mathbf{y}} \left(\bigotimes_{i=0}^{d} |i, x_i\rangle |y_i\rangle\right) \otimes |w_{j,\mathbf{x},\mathbf{y}}\rangle,$$

then for any $\mathcal{F} \in \mathbf{SHUF}(f,d)$,

$$\sum_j \lambda_j \sum_{\mathbf{x}:x_d \in S_d(\mathcal{F}),\mathbf{y}} v_{j,\mathbf{x},\mathbf{y}}^2 \leq \exp(-\lambda n'/2) + \exp(-Cn').$$

We see that $O_{f,d} \otimes \mathrm{Id}$ maps $|\phi_j\rangle\langle\phi_j|$ to

$$\mathbb{E}_{\mathcal{F}\sim\mathcal{D}(f,d)}\Bigg[\sum_{\mathbf{x},\mathbf{x}',\mathbf{y},\mathbf{y}'} v_{j,\mathbf{x},\mathbf{y}} v_{j,\mathbf{x}',\mathbf{y}'} \bigotimes_{i=0}^{d-1} |x_i, y_i \oplus f_i(x_i)\rangle\langle x_i', y_i' \oplus f_i(x_i)|$$
$$\otimes \left(|x_d, y_d \oplus f_d(x_d)\rangle \cdot \mathbb{1}[x_d \in S_d(\mathcal{F})] + |x_d, y_d\rangle \cdot \mathbb{1}[x_d \notin S_d(\mathcal{F})]\right)$$
$$\left(\langle x_d', y_d' \oplus f_d(x_d')| \cdot \mathbb{1}[x_d' \in S_d(\mathcal{F})] + \langle x_d', y_d'| \cdot \mathbb{1}[x_d' \notin S_d(\mathcal{F})]\right) \otimes |w_{j,\mathbf{x},\mathbf{y}}\rangle\langle w_{j,\mathbf{x}',\mathbf{y}'}|\Bigg]$$

Noting that the only dependence on f in the above expression is in the definition of f_d, we see that for any $f, f' : \{0,1\}^n \to \{0,1\}^n$,

$$(O_{f,d}\otimes\mathrm{Id} - O_{f',d}\otimes\mathrm{Id})[|\phi_j\rangle\langle\phi_j|] = \mathbb{E}_{\mathcal{F}\sim\mathcal{D}(f,d)}\Bigg[\sum_{\mathbf{x},\mathbf{x}',\mathbf{y},\mathbf{y}'} v_{j,\mathbf{x},\mathbf{y}} v_{j,\mathbf{x}',\mathbf{y}'} \bigotimes_{i=0}^{d-1} |x_i, y_i \oplus f_i(x_i)\rangle\langle x_i', y_i' \oplus f_i(x_i)|$$
$$\otimes \Big\{ \langle x_d, y_d \oplus f_d(x_d)|\,|x_d', y_d' \oplus f_d'(x_d')\rangle \cdot \mathbb{1}[x_d, x_d' \in S_d(\mathcal{F})]$$
$$+ \langle x_d, y_d \oplus f_d(x_d)|\,|x_d', y_d'\rangle \cdot \mathbb{1}[x_d \in S_d(\mathcal{F}), x_d' \notin S_d(\mathcal{F})]$$
$$+ \langle x_d, y_d|\,|x_d', y_d' \oplus f_d'(x_d')\rangle \cdot \mathbb{1}[x_d \notin S_d(\mathcal{F}), x_d' \in S_d(\mathcal{F})]\Big\} \otimes |w_{j,\mathbf{x},\mathbf{y}}\rangle\langle w_{j,\mathbf{x}',\mathbf{y}'}|\Bigg]$$

where we define $f_d'(x) = f'((f_{d-1} \circ \cdots \circ f_0)^{-1}(x))$ for $x \in S_d(\mathcal{F})$ and $f_d'(x) = \mathbf{0}$ otherwise. This is a mixed state of rank at most 2, so its trace norm is at most $\sqrt{2}$ times its Frobenius norm, which we can bound via triangle inequality by

$$\mathbb{E}_{\mathcal{F}\sim\mathcal{D}(f,d)}\left[2\sqrt{\sum_{\substack{\mathbf{x},\mathbf{x}',\mathbf{y},\mathbf{y}':\\ x_d\in\Omega \text{ or } x_d'\in\Omega}} v_{j,\mathbf{x},\mathbf{y}}^2 v_{j,\mathbf{x}',\mathbf{y}'}^2} \leq 2\sqrt{1-\left(1-\sum_{\mathbf{x}:x_d\in S_d,\mathbf{y}} v_{j,\mathbf{x},\mathbf{y}}^2\right)^2} \leq 2\sqrt{2\sum_{\mathbf{x}:x_d\in S_d,\mathbf{y}} v_{j,\mathbf{x},\mathbf{y}}^2}\right].$$

Letting $j \sim \lambda$ denote sampling from the distribution which places mass λ_j on index j, we conclude that

$$\begin{aligned}
\|(O_{f,d}\otimes\mathrm{Id} - O_{f',d}\otimes\mathrm{Id})[D_\lambda^{\otimes n''}[\sigma]]\|_{\mathsf{tr}} &\leq 4\mathbb{E}_{j\sim\lambda,\mathcal{F}\sim\mathcal{D}(f,d)}\left[\sqrt{\sum_{\mathbf{x}:x_d\in S_d(\mathcal{F}),\mathbf{y}} v_{j,\mathbf{x},\mathbf{y}}^2}\right] \\
&\leq 4\mathbb{E}_{\mathcal{F}\sim\mathcal{D}(f,d)}\left[\sqrt{\mathbb{E}_{j\sim\lambda}\left[\sum_{\mathbf{x}:x_d\in S_d(\mathcal{F}),\mathbf{y}} v_{j,\mathbf{x},\mathbf{y}}^2\right]}\right] \\
&\leq 4(\exp(-\lambda n'/4) + \exp(-Cn'/2) + 2^{-cdn}) = \exp(-\Omega(\lambda d n)).
\end{aligned}$$

The lemma follows by taking the channels $\mathcal{E}$ in Lemma 3.B.1 to be $\mathbb{E}_{f\ \text{2-to-1}}[(O_{f,d}\otimes\mathrm{Id})\circ D_\lambda^{\otimes n''}]$ and $\mathbb{E}_{f\ \text{1-to-1}}[(O_{f,d}\otimes\mathrm{Id})\circ D_\lambda^{\otimes n''}]$. ☐

Proof of Theorem 8..2. Let $\mathcal{T}$ be the learning tree corresponding to a NISQ_λ algorithm that makes at most N classical queries to f or quantum oracle queries to $O_{f,d}$. By Lemma 3.A.1 and Lemma 8..3, if we replace every noisy quantum circuit A in the tree with a noisy quantum circuit A' that makes queries to $O_{g,d}$ for $g : \{0,1\}^n \to \{0,1\}^n$ the identity function, then the new distribution over the leaves of $\mathcal{T}$ is at most $N^2 \exp(-\Omega(\lambda dn))$-far in total variation from the original distribution $p_{O_{f,d}}$; for $N = \exp(o(\lambda dn))$, this quantity is $o(1)$. For convenience, denote this new distribution p'_f.

To apply Lemma 3.A.2, we wish to bound $d_{\mathrm{TV}}(\mathbb{E}_{f \text{ 1-to-1}}[p'_f], \mathbb{E}_{f \text{ 2-to-1}}[p'_f])$. But note that because the noisy quantum circuits A' in the new learning tree are independent of the underlying function f, the learning tree is simply implementing a randomized classical query algorithm. As in the proof of Theorem 4.B.1, if p'^r_f denotes the distribution p'_f conditioned on some internal randomness r of the algorithm, it suffices to bound $\sup_r d_{\mathrm{TV}}(\mathbb{E}_{f \text{ 1-to-1}}[p'_f], \mathbb{E}_{f \text{ 2-to-1}}[p'_f])$. The bound for this can be proven in an identical fashion as in Theorem 4.B.1, so we conclude that this quantity is $o(1)$ if there are $o(2^{n/2})$ classical queries along any root-to-leaf path of $\mathcal{T}$. □

Supplementary Note 9 – Deferred Proofs

9.A Proof of Lemma 5.C.1

Given $S \subseteq [n]$ and mixed n-qubit state σ, let $\sigma|_S$ denote the restriction of σ to the subsystem indexed by S. That is,

$$\sigma_{!S} = \left(\mathrm{tr}(\sigma|_{S^c}) \cdot I/2^{|S^c|}\right) \otimes \sigma|_S. \tag{22}$$

Proof. Von Neumann entropy is invariant under unitary transformation, so it suffices to show that for any mixed state σ, $I(D_\lambda[\sigma]) \leq (1-\lambda) \cdot I(\sigma)$. Because $D_\lambda[\sigma] = \mathbb{E}_{S\sim\mu}[\sigma_{!S}]$ for μ the distribution over $S \subseteq [n]$ which includes each element of $[n]$ independently with probability λ. So by concavity of entropy, additivity of entropy for tensor products, and (22),

$$I(D_\lambda[\sigma]) \leq \sum_{k=0}^{n} \lambda^{n-k}(1-\lambda)^k \sum_{S\subset[n]:|S|=k} I(\sigma|_S) \leq \sum_{k=0}^{n} \binom{n}{k} \frac{k}{n} \lambda^{n-k}(1-\lambda)^k = (1-\lambda) I(\sigma),$$

where in the last step we used Lemma 9.A.1 below. □

The proof above uses the following fact:

Lemma 9.A.1 (Lemma 7 from [22])**.** *For any density matrix σ on n qubits and any $0 \leq k < n$,*

$$\binom{n}{k}^{-1} \sum_{S\subset[n]:|S|=k} I(\sigma|_S) \leq \frac{k}{n} I(\sigma).$$

9.B Proof of Lemma 4.B.1

Proof. Given $S \subseteq [n]$ and $z \in \{0,1\}^{|S|}, y \in \{0,1\}^{n-|S|}$, let $z \circ_S y \in \{0,1\}^n$ denote the string whose i-th entry is z_i if $i \in S$ and y_i otherwise.

Let $|\psi\rangle = \sum_{x\in\{0,1\}^{n'}} c_x |x\rangle$, and for convenience let $\rho_\psi \triangleq D_\lambda^{\otimes n'}[|\psi\rangle\langle\psi|]$. For any $S \subseteq [n']$, we have

$$\mathrm{tr}_S(|\psi\rangle\langle\psi|) = \sum_{y,y'\in\{0,1\}^{2n-|S|}} \left(\sum_{w\in\{0,1\}^{|S|}} c_{w\circ_S y} c_{w\circ_S y'}\right) |y\rangle\langle y'|.$$

For convenience, denote the coefficient $\sum_w c_{w\circ_S y} c_{w\circ_S y'}$ by $C^S_{y,y'}$. If S is sampled by including every $i \in [n']$ independently with probability $\lambda/2$, then

$$\rho_\psi = \mathbb{E}_S\Big[\sum_{z\in\{0,1\}^{|S|}, y,y'\in\{0,1\}^{n'-|S|}} \frac{1}{2^{|S|}} |z\rangle\langle z| \otimes_S C^S_{y,y'} |y\rangle\langle y'|\Big].$$

Note that for any $x \in \Omega$,

$$\begin{aligned}
\langle x|\,\rho_\psi\,|x\rangle &= \mathbb{E}_{S\sim\mu}\Big[\frac{1}{2^{|S|}}\sum_{z,y} C^S_{y,y}\cdot \mathbb{1}[z\circ y = x]\Big] \\
&= \mathbb{E}_{S\sim\mu}\Big[\frac{1}{2^{|S|}} C^S_{x_{[n']\backslash S}, x_{[n']\backslash S}}\Big] \\
&= \mathbb{E}_{S\sim\mu}\Big[\frac{1}{2^{|S|}}\sum_{w\in\{0,1\}^{|S|}} c^2_{w\circ_S x_{[n]\backslash S}}\Big].
\end{aligned}$$

Note that this expression is precisely $\Pr_{a\sim D,\tilde{a}}[\tilde{a} = x]$, so $\mathrm{tr}(\Pi\rho_\psi)$ is the probability that $\tilde{a} \in \Omega$. ☐

Supplementary Note 10 – Simon's Problem and Noisy Parity

In this section we describe a path towards separating NISQ and BPP using the original Simon's problem, i.e. without modifications based on quantum error-correcting codes like in Section 4.A. We begin by recalling the problem of learning parity with noise and a classical result on the computational complexity of this problem.

Definition 10..1. *For $\eta \in [0, 1/2)$ and $n \in \mathbb{N}$, an instance of* noisy parity with noise rate η *is given by an unknown string $s \in \{0,1\}^n$ and samples $(x_1, y_1), \ldots, (x_N, y_N)$ where each x_i is sampled uniformly from $\{0,1\}^n$ and each y_i is independently either $\langle x_i, s\rangle \bmod 2$ with probability $1-\eta$ or $1 - \langle x_i, s\rangle \bmod 2$ otherwise. We say that an algorithm solves noisy parity with noise rate η with n samples if, given such an instance, it recovers S with probability at least $2/3$ over its internal randomness and the randomness of the samples.*

While is widely conjectured (see e.g. [35]) that this problem is computationally hard, the following seminal result implies that it is possible to do somewhat better than brute force:

Theorem 10..1 (Theorem 2 and Section 3.3 from [36])**.** *If the unknown string s in noisy parity is promised to be a subset of the first $\mathcal{O}(\log n \cdot \log\log n)$ bits, then there is an algorithm for noisy parity with noise rate η which has runtime and sample complexity* $\mathrm{poly}(n)\cdot(1-2\eta)^{-\sqrt{\log n}}$.

Now consider Simon's problem on n qubits, with the promise that the unknown period $s \in \{0,1\}^n$ is supported on the first $k \triangleq \mathcal{O}(\log n \cdot \log\log n)$ bits and has Hamming weight at most $\log n$. Note that because there are $\binom{\log n\cdot\log\log n}{\log n}$ such periods, the complexity of this special case of Simon's problem for classical algorithms is super-polynomial in n.

We now sketch an argument showing that under a plausible strengthening of Theorem 10..1, there is a NISQ algorithm that solves this special case of Simon's efficiently. Note that if one runs Simon's algorithm restricted to these qubits using a λ-noisy quantum circuit with $\lambda \in (0,1)$ an absolute constant, then with probability $(1-\lambda)^{\mathcal{O}(\log n)} \geq 1/\mathrm{poly}(n)$ none of the qubits $i \in [n]$ for which $s_i \neq 0$ will be decohered over the course of Simon's algorithm, in which case the classical string $x \in \{0,1\}^n$ satisfies $\langle x, s\rangle \equiv 0 \bmod 2$. Otherwise, if one of the first k qubits gets decohered at any point, the classical string x satisfies $\langle x, s\rangle \equiv 0 \bmod 2$ with probability $1/2$. In particular,

if one runs this noisy quantum circuit N times, the result is an instance of noisy parity with noise rate η satisfying $1 - 2\eta \geq 1/\mathrm{poly}(n)$. In particular, if one could improve the dependence on η in Theorem 10..1 from $(1-2\eta)^{-\sqrt{\log n}}$ to $\mathrm{poly}(1/(1-2\eta))$, then we would find that NISQ algorithms can efficiently solve this special case of Simon's problem. Formally, we have concluded the following:

Theorem 10..2. *Suppose there is an algorithm for noisy parity with noise rate η, where the unknown string s is promised to be a subset of the first $\mathcal{O}(\log n \cdot \log\log n)$ bits, with runtime $\mathrm{poly}(n, (1-2\eta)^{-1})$. Then for O the oracle for Simon's problem under the promise that the unknown period is supported on the first $\mathcal{O}(\log n \cdot \log\log n)$ bits and has Hamming weight at most $\log n$, $\mathsf{BPP}^O \subsetneq \mathsf{NISQ}^O$.*

References

[1] Siddharth Muthukrishnan, Tameem Albash, and Daniel A. Lidar. Sensitivity of quantum speedup by quantum annealing to a noisy oracle. *Physical Review A*, 99(3):032324, 2019.

[2] Neil Shenvi, Kenneth R. Brown, and K. Birgitta Whaley. Effects of a random noisy oracle on search algorithm complexity. *Physical Review A*, 68(5):052313, 2003.

[3] Gui Lu Long, Yan Song Li, Wei Lin Zhang, and Chang Cun Tu. Dominant gate imperfection in grover's quantum search algorithm. *Physical Review A*, 61(4):042305, 2000.

[4] Oded Regev and Liron Schiff. Impossibility of a quantum speed-up with a faulty oracle. In *International Colloquium on Automata, Languages, and Programming*, pages 773–781. Springer, 2008.

[5] Andris Ambainis, Artūrs Bačkurs, Nikolajs Nahimovs, and Alexander Rivosh. Grover's algorithm with errors. In *International Doctoral Workshop on Mathematical and Engineering Methods in Computer Science*, pages 180–189. Springer, 2012.

[6] Andrew W. Cross, Graeme Smith, and John A. Smolin. Quantum learning robust against noise. *Physical Review A*, 92(1):012327, 2015.

[7] Alex B Grilo, Iordanis Kerenidis, and Timo Zijlstra. Learning-with-errors problem is easy with quantum samples. *Physical Review A*, 99(3):032314, 2019.

[8] Matthias C Caro. Quantum learning boolean linear functions wrt product distributions. *Quantum Information Processing*, 19(6):1–41, 2020.

[9] Ansis Rosmanis. Hybrid quantum-classical search algorithms. *arXiv:2202.11443*, 2022.

[10] Nai-Hui Chia, Kai-Min Chung, and Ching-Yi Lai. On the need for large quantum depth. In *Proceedings of the 52nd Annual ACM SIGACT Symposium on Theory of Computing*, pages 902–915, 2020.

[11] Matthew Coudron and Sanketh Menda. Computations with greater quantum depth are strictly more powerful (relative to an oracle). In *Proceedings of the 52nd Annual ACM SIGACT Symposium on Theory of Computing*, pages 889–901, 2020.

[12] Xiaoming Sun and Yufan Zheng. Hybrid decision trees: Longer quantum time is strictly more powerful. *arXiv preprint arXiv:1911.13091*, 2019.

[13] Daniel Shapira, Shay Mozes, and Ofer Biham. Effect of unitary noise on Grover's quantum search algorithm. *Physical Review A*, 67(4):042301, 2003.

[14] B. Pablo-Norman and M. Ruiz-Altaba. Noise in grover's quantum search algorithm. *Physical Review A*, 61(1):012301, 1999.

[15] Frédéric Magniez, Ashwin Nayak, Jérémie Roland, and Miklos Santha. Search via quantum walk. In *Proceedings of the thirty-ninth annual ACM symposium on Theory of computing*, pages 575–584, 2007.

[16] Daniel Stilck França and Raul Garcia-Patron. Limitations of optimization algorithms on noisy quantum devices. *Nature Physics*, 17(11):1221–1227, 2021.

[17] Kunal Sharma, Sumeet Khatri, Marco Cerezo, and Patrick J. Coles. Noise resilience of variational quantum compiling. *New Journal of Physics*, 22(4):043006, 2020.

[18] Enrico Fontana, Nathan Fitzpatrick, David Muñoz Ramo, Ross Duncan, and Ivan Rungger. Evaluating the noise resilience of variational quantum algorithms. *Physical Review A*, 104(2):022403, 2021.

[19] Wim Lavrijsen, Ana Tudor, Juliane Müller, Costin Iancu, and Wibe De Jong. Classical optimizers for noisy intermediate-scale quantum devices. In *2020 IEEE International Conference on Quantum Computing and Engineering (QCE)*, pages 267–277. IEEE, 2020.

[20] Samson Wang, Enrico Fontana, Marco Cerezo, Kunal Sharma, Akira Sone, Lukasz Cincio, and Patrick J Coles. Noise-induced barren plateaus in variational quantum algorithms. *Nature communications*, 12(1):1–11, 2021.

[21] Adam Bouland, Bill Fefferman, Zeph Landau, and Yunchao Liu. Noise and the frontier of quantum supremacy. In *2021 IEEE 62nd Annual Symposium on Foundations of Computer Science (FOCS)*, pages 1308–1317. IEEE, 2022.

[22] Dorit Aharonov, Michael Ben-Or, Russell Impagliazzo, and Noam Nisan. Limitations of noisy reversible computation. *quant-ph/9611028*, 1996.

[23] Sitan Chen, Jordan Cotler, Hsin-Yuan Huang, and Jerry Li. Exponential separations between learning with and without quantum memory. *To appear in FOCS*, 2021.

[24] Dorit Aharonov and Michael Ben-Or. Fault-tolerant quantum computation with constant error. In *Proceedings of the twenty-ninth annual ACM symposium on Theory of computing*, pages 176–188, 1997.

[25] Daniel R. Simon. On the power of quantum computation. *SIAM Journal on Computing*, 26(5):1474–1483, 1997.

[26] Christof Zalka. Grover's quantum searching algorithm is optimal. *Physical Review A*, 60(4):2746, 1999.

[27] Ethan Bernstein and Umesh Vazirani. Quantum complexity theory. *SIAM Journal on Computing*, 26(5):1411–1473, 1997.

[28] Hsin-Yuan Huang, Richard Kueng, and John Preskill. Information-theoretic bounds on quantum advantage in machine learning. *Physical Review Letters*, 126(19):190505, 2021.

[29] Hsin-Yuan Huang, Michael Broughton, Jordan Cotler, Sitan Chen, Jerry Li, Masoud Mohseni, Hartmut Neven, Ryan Babbush, Richard Kueng, John Preskill, et al. Quantum advantage in learning from experiments. *Science*, 376(6598):1182–1186, 2022.

[30] Scott Aaronson. Shadow tomography of quantum states. In *Proceedings of the 50th Annual ACM SIGACT Symposium on Theory of Computing*, pages 325–338, 2018.

[31] Scott Aaronson and Guy N. Rothblum. Gentle measurement of quantum states and differential privacy. In *Proceedings of the 51st Annual ACM SIGACT Symposium on Theory of Computing*, pages 322–333, 2019.

[32] Costin Bădescu and Ryan O'Donnell. Improved quantum data analysis. In *Proceedings of the 53rd Annual ACM SIGACT Symposium on Theory of Computing*, pages 1398–1411, 2021.

[33] Hsin-Yuan Huang, Richard Kueng, and John Preskill. Predicting many properties of a quantum system from very few measurements. *Nature Physics*, 16(10):1050–1057, 2020.

[34] Hsin-Yuan Huang, Steven T. Flammia, and John Preskill. Foundations for learning from noisy quantum experiments. *arXiv:2204.13691*, 2022.

[35] Avrim Blum, Merrick Furst, Michael Kearns, and Richard J Lipton. Cryptographic primitives based on hard learning problems. In *Annual International Cryptology Conference*, pages 278–291. Springer, 1993.

[36] Avrim Blum, Adam Kalai, and Hal Wasserman. Noise-tolerant learning, the parity problem, and the statistical query model. *Journal of the ACM (JACM)*, 50(4):506–519, 2003.
